# Predicting Metastasis Risk in Pancreatic Neuroendocrine Tumors Using Deep Learning Image Analysis

**DOI:** 10.3389/fonc.2020.593211

**Published:** 2021-02-25

**Authors:** Sergey Klimov, Yue Xue, Arkadiusz Gertych, Rondell P. Graham, Yi Jiang, Shristi Bhattarai, Stephen J. Pandol, Emad A. Rakha, Michelle D. Reid, Ritu Aneja

**Affiliations:** ^1^ Department of Biology, Georgia State University, Atlanta, GA, United States; ^2^ Department of Mathematics and Statistics, Georgia State University, Atlanta, GA, United States; ^3^ Department of Pathology, Northwestern University, Chicago, IL, United States; ^4^ Department of Surgery, Cedars-Sinai Medical Center, Los Angeles, CA, United States; ^5^ Department of Pathology and Laboratory Medicine, Cedars-Sinai Medical Center, Los Angeles, CA, United States; ^6^ Faculty of Biomedical Engineering, Silesian University of Technology, Zabrze, Poland; ^7^ Department of Laboratory Medicine and Pathology, Mayo Clinic, Rochester, MN, United States; ^8^ Department of Medicine, Cedars-Sinai Medical Center, Los Angeles, CA, United States; ^9^ Department of Cellular Pathology, University of Nottingham, Nottingham, United Kingdom; ^10^ Department of Pathology, Emory University, Atlanta, GA, United States

**Keywords:** metastasis risk assessment, deep learning, histological image analysis, pancreatic neuroendocrine tumors, computational pathology

## Abstract

**Background:**

The prognosis of patients with pancreatic neuroendocrine tumors (PanNET), the second most common type of pancreatic cancer, varies significantly, and up to 15% of patients develop metastasis. Although certain morphological characteristics of PanNETs have been associated with patient outcome, there are no available morphology-based prognostic markers. Given that current clinical histopathology markers are unable to identify high-risk PanNET patients, the development of accurate prognostic biomarkers is needed. Here, we describe a novel machine learning, multiclassification pipeline to predict the risk of metastasis using morphological information from whole tissue slides.

**Methods:**

Digital images from surgically resected tissues from 89 PanNET patients were used. Pathologist-annotated regions were extracted to train a convolutional neural network (CNN) to identify tiles consisting of PanNET, stroma, normal pancreas parenchyma, and fat. Computationally annotated cancer or stroma tiles and patient metastasis status were used to train CNN to calculate a region based metastatic risk score. Aggregation of the metastatic probability scores across the slide was performed to predict the risk of metastasis.

**Results:**

The ability of CNN to discriminate different tissues was high (per-tile accuracy >95%; whole slide cancer regions Jaccard index = 79%). Cancer and stromal tiles with high evaluated probability provided F1 scores of 0.82 and 0.69, respectively, when we compared tissues from patients who developed metastasis and those who did not. The final model identified low-risk (n = 76) and high-risk (n = 13) patients, as well as predicted metastasis-free survival (hazard ratio: 4.71) after adjusting for common clinicopathological variables, especially in grade I/II patients.

**Conclusion:**

Using slides from surgically resected PanNETs, our novel, multiclassification, deep learning pipeline was able to predict the risk of metastasis in PanNET patients. Our results suggest the presence of prognostic morphological patterns in PanNET tissues, and that these patterns may help guide clinical decision making.

## Introduction

Pancreatic neuroendocrine tumors (PanNETs) represent a subset of pancreatic neoplasms. Though traditionally considered a rare subset, recent studies suggest that PanNETs comprise approximately 10% of all pancreatic malignancies (1). PanNETs originate from neuroendocrine epithelial cells, often resembling the cells of the islets of Langerhans. Notably, PanNETs can also secrete hormones (e.g., insulin) into the bloodstream; these hormones producing PanNETs are known as functional PanNETs. According to the WHO classification system, PanNETs are classified as well-differentiated (WDNETs or “ordinary”) or poorly differentiated (PDNEC) subtypes. Recent evidence suggests that these tumors are not in a continuum and should, thus, be regarded separately (2, 3). PDNEC risk stratification is less ambiguous, and PDNEC patients often have poor outcomes (median survival typically under 2 years) (4). In contrast, the overall survival of patients with WDNET (hereinafter referred to as PanNET) is relatively high (10-year survival rate of 60%–70%); nevertheless the risk of metastasis is high (up to 15%), even for small lesions (4).

The lack of robust biomarkers remains the most significant clinical hurdle for accurate prognosis prediction in PanNET patients. The mitotic count and the Ki-67 index are currently the only prognostic biomarkers routinely used clinically (5, 6). Nonetheless, these two indexes are prone to quantification errors. For instance, cells expressing mitotic mimics (such as cells undergoing pyknosis) compromise mitotic count accuracy, and depending on the counting methodology used Ki-67 can show poor concordance (7). In addition to technical issues in these scoring systems, the lack of consensus in scoring cutoffs further limits their ability to provide accurate patient stratification (8). Recently developed models based on Ki-67 scoring (5) or linear pathological combinative approaches (6) failed to improve metastasis risk prediction in PanNET patients (5, 6). Thus, robust and accurate models to predict the risk of metastasis are unmet clinical needs.

Histologic alterations, such as necrosis, variations in nuclear shape (atypia), chromatin clumping, and reduction in the tumor stroma, are high-risk components in PanNETs (9). Despite significant variation in the morphological characteristics of PanNETs and recent reports suggesting a link between morphological features of PanNETs and aggressive behavior (9–11), there are no morphology-based tools for outcome prediction.

**Figure 1:**
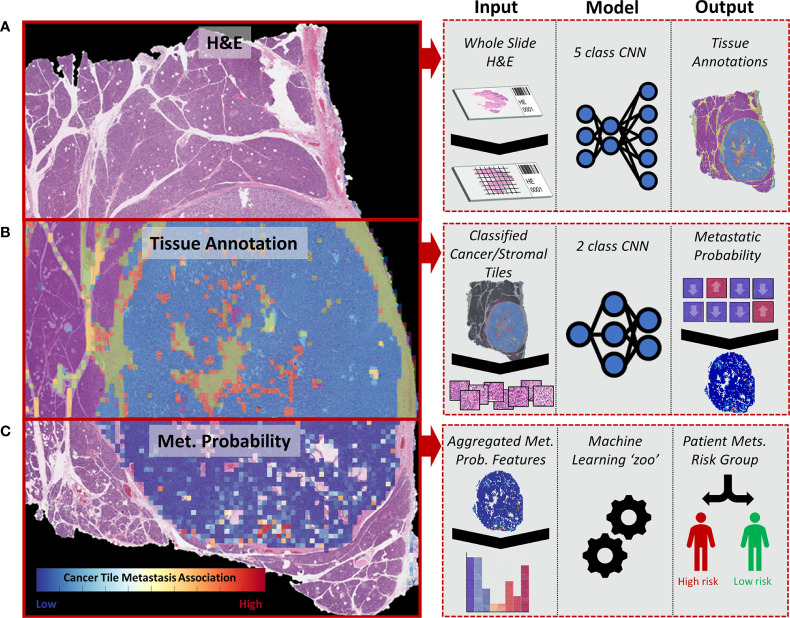
Diagram representing the whole-slide image (WSI) processing pipeline used to stratify PanNET patients into high- and low-risk metastasis groups. **(A)** H&E-stained tissues from surgical resections are split into image tiles using a sliding window approach and are classified into different tissue types: cancer (stromal poor/clearly delineated), cancer (stroma-rich), stroma without cancer, normal parenchymal, and fat. **(B)** Image tiles classified as cancer and adjacent stromal regions are further classified into “metastasis” or “non-metastasis” groups depending on the presence of metastatic lesions in the patient. **(C)** The tile-based metastasis association scores from a WSI are used to determine a set of WSI features fitted to a collection of machine learning algorithms (“zoo”) to determine the overall risk of metastasis (high vs. low).

Herein, we present a novel convolutional neural network (CNN)-based multiclassification pipeline for morphological analysis of whole-slide images (WSIs) ultimately tailored toward predicting outcome. CNNs have emerged as a powerful tool to identify morphologically distinct areas on digitized slides (12) and correlate image patterns, even subtle ones, to patient prognosis (13). The models presented here provide a machine learning-based approach to identify relevant tissue regions within whole tissue slides and predict the risk of metastasis based on the morphological features of PanNET and the surrounding stroma (**Figure 1**).

## Methods

### Study Population

Tissue samples were obtained from surgical resections of PanNET patients treated at Emory University hospital between 2002 and 2017 (**Table 1**). Patients presenting with metastasis during surgery and those lost to follow-up within a year after surgery were omitted from the analysis. In total, we analyzed samples from 89 cases in the study, 20% of which developed metastasis (detected by biopsy or imaging). Patient records were reviewed to obtain follow-up, demographic, and clinicopathological data. Grade was computed through the Ki-67 index, when available, based on the WHO criteria thresholds (14). Most patients (77.5%) were categorized as Grade I/II (Ki-67<20) and considered “low grade.” Metastasis-free survival was measured from the time of surgery to the time of metastasis or last follow-up.

**Table 1:** Clinicopathological characteristics of the PanNET cohort.

Patient Clinicopathological Characteristics	
Baseline characteristic	Total (N = 89)
**Patient age**	
Median Age (range), years	56 (19–82)
Age <50, n (%)	25 (28.1)
Age> = 50, n (%)	53 (59.6)
Missing	11 (12.3)
**Tumor size**	
Median Size (range), cm	3 (0.6–11)
Size <2.0, n (%)	34 (37.1)
Size>=2.0, n (%)	43 (48.3)
Missing	13 (14.6)
**Sex, n (%)**	
Male	35 (39.3)
Female	44 (48.3)
Missing	11 (12.4)
**Metastasis status, n (%)**	
Recurrence free	71 (80.0)
Recurred	18 (20.0)
Missing	0 (0.00)
**Grade, n (%)**	
1	58 (65.2)
2	11 (12.4)
3	1 (1.1)
Missing	19 (21.3)

### Tumor Slide Selection

Representative (based on tumor tissue and morphological variability) H&E-stained whole slides were selected from archived formalin-fixed paraffin-embedded tumors. Our analyses included an average of 1.17 slides per patient, with review and scanning of a single representative slide in the majority (n = 75) of patients. The slides were digitized using Aperio AT Turbo scanner (Leica Biosystems, Vista, CA) with high-resolution image settings (40×, magnification 0.24 µm/pixel size). Images of low quality were re-scanned or omitted from further analysis.

### Automated Full Slide Annotation

We used the pre-trained GoogLeNet (Inception V1) CNN model (15) with a modified terminal softmax layer and classified tissues into cancer (stromal poor/clearly delineated), cancer (stroma-rich), stroma without cancer, normal parenchymal, and fat. Pathologist-annotated ground-truth regions from 11 partially annotated slides for each class were extracted using the MATLAB Image Labeler (**Supplementary Figure 1**) (16). Aside from the cancer/stroma class determined by any cancer cluster within a stromal rich region (fibrous stroma, fibrovascular stroma, fibrous septa, or loose or hyalinized stroma), training tiles came almost exclusively from non-mixed ground-truth regions. The intersection of annotations (edges) was labeled with the predominant class in the image tile.

WSIs were down-sampled four times for tissue classification, allowing for computational feasibility while providing clear visual discrimination of the gross scaled annotation regions. Each tile (for both training and classification) was color-normalized (17) to ensure H&E staining consistency and improve machine learning performance (18). Additionally, a thorough augmentation of training tiles (12) was performed to improve the robustness of the CNN classifier and reduce the risk of overfitting, without compromising the image quality (19). For CNN training data, tiles were adjusted for image orientation and hue/blur/noise/contrast as previously described (12), to expand the training set of tiles by a factor of 46. This process resulted in a final training set of 466,072 tiles. CNN training was optimized using the stochastic gradient descent (SGD) algorithm with a momentum of 0.9, batch size of 35 tiles, and learning rate of 1e-4. The training tiles were reshuffled after each epoch, and model accuracy was measured at the end of each epoch. Model training was continued until the multiclass accuracy for each label was over 99%. The trained model was then tested using tiles from four external slides. Overlapping tiles (50%) from the validation slides were extracted (without augmentation) to produce a validation cohort of 42,976 tiles.

WSI annotation was performed by fully partitioning slides into non-overlapping 150 × 150 tiles, with background “non-tissue” regions omitted from classification. These segmented tiles were independently classified with the trained CNN to produce a five-dimensional output, with each dimension representing the probability of the tiles belonging to one of the five classes. To assess the WSI classification performance, we overlaid CNN-annotated cancer areas onto pathologist-annotated areas and calculated the Jaccard index.

### Metastasis Association Classifier

After WSI annotation, two GoogLeNet (Inception V1) classifiers were trained to predict metastasis for tiles classified as cancer or stroma. For classifier training, we selected only tiles classified with a high probability (95%) as either cancer or stroma. Through this tile filtering step, we excluded 20% of all stromal and cancer tiles. Subsequently, WSIs (89 patients/104 slides) were subjected to a five-fold cross-validation. WSIs from different tumor blocks of the same patient (n = 14) were kept together in the same training or validation fold to ensure that each validation contained tiles from WSIs that the classifier was not trained with. During training, the patient’s distant metastasis status was used as the ground-truth label for all tiles selected from the respective WSIs. Each metastasis association classifier was trained for 15 epochs (using SGD with a momentum of 0.9 and an initial learning rate of 0.0001 decreased by a factor of 0.1 after five epochs) and subsequently used for the respective validation fold. The risk of overfitting to the training data was reduced by applying an L2 regularization of 0.0001. The predicted metastasis association labels (and softmax class probability scores) for tiles within each validation fold were used for further analysis. Confusion matrices (comparing true vs. predicted labels of tiles labeled with metastasis vs. no metastasis) were obtained at different CNN output probability scores (starting at the classification default of >50% to restricting analysis to > 99.99% class probability) to assess the classification performance of the tile probability score. Moreover, we performed uniform manifold approximation and projection (UMAP) for dimension reduction (20, 21) using pooling layer features before the final CNN layer to further validate the discriminatory ability of these classifiers. This reduction step reduced the vector of activations from 1024 to 2. Bivariate kernel density estimators were fitted with a Gaussian kernel to visually determine UMAP cluster densities.

### WSI Feature Extraction and Metastasis Prediction

Metastasis probabilities for cancer and stromal tiles were combined to obtain a WSI risk score. Tiles without sufficient, directly adjacent, similarly annotated neighbors (tissues) were considered artifacts and filtered out (n = 8 for cancer; n = 3 for stroma). The distribution of the metastasis association score (0–100%) for the remaining tiles within each slide was the basis for the extraction of 150 “full slide” features (75 from cancer and 75 from stromal; **Supplementary Table 1**). These features were derived from histogram metrics of both individual image tiles within the WSI and after aggregation within a 10 × 10 tile area. Metrics were derived from the metastasis risk score histogram and included the statistical moments (mean, standard deviation, skewness, and kurtosis) and the tile counts/proportions. These metrics were obtained for each WSI within a) all metastasis probability tile distributions and b) within only the high (>90%) and low (<10%) probability tails of the tile distribution. The features derived from 10 × 10 tile areas were bin counts of “spatially clustered” metastasis-associated groups assuming that clusters of high-risk areas possess potential prognostic value beyond single regions. Missing values were imputed using Multivariate Imputation by Chained Equations (mice) with 10 iterations (22).

The 150 whole-slide features extracted from WSIs were grouped into three subsets: 1) cancer-only features 2) stromal-only features and 3) stromal and cancer features. These feature subsets were used as input variables for 18 different machine learning models (**Supplementary Table 2**) alongside the patient’s metastatic status (n = 18 metastasis, n = 71 no metastasis) as the labels. The models were trained through leave-one-out cross-validation (LOOCV), wherein each left-out set composed of all the slides from a single patient. Patients with multiple slides (n = 14) were given a “high-risk” prediction if any of their slides were predicted to metastasize. An ensembling approach was also tested by combining the outputs of the models trained with stromal features to those trained with cancer features; patients were considered at high-risk if either approach predicted metastasis.

To improve accuracy (23) and reduce data dimensionality, we performed a filtering-based feature selection. For each training fold within the leave-one-out cross-validation, a two-sample (Welch) t-test was performed comparing all features of patients who developed metastasis to those who did not. Each t-test provided the feature with a t-score which signified the magnitude of the mean difference between that feature with patients who metastasized versus those which did not. Multiple filtering thresholds were tested to optimize the feature set by removing features which did not have large enough t-scores, or significance. This model was further analyzed univariately using Kaplan-Meier survival analysis and multivariately (alongside tumor size, patient age, and sex) using Cox Regression on all patients and low (I/II) grade patients only. SHAP (Shapley additive explanation) values were used to interpret the output of the selected models (24, 25) and assess feature importance (26). Calculation of SHAP values was performed for each left-out test set and then aggregated because of the LOOCV nature of the results.

### Pipeline Generalization

To analyze the pipeline’s generalizability with a stronger control for overfitting, a nested LOOCV approach (**Supplementary Figure 2**) was tested. The nested LOOCV approach consists of an outer loop which splits the data into a training set and a patient left out test set, and an inner loop with a 10-fold CV for feature selection through t-test filtering. For the inner loop, for each CV, a t-test is applied to all features to determine the magnitude of the average difference (t-score) for those features coming from slides of patients who metastasized versus those that did not. Sorting features by this t-score allowed for a filtering approach which, at each increasing t-score threshold, retained increasingly significant variables. By progressively increasing the t-score threshold for filtering, and then training the inner loop CV models, an optimized feature (or t-score threshold) set can be selected by identifying what model resulted in the best average performance (through log-loss) in the inner CV loop.

The random forest in the outer loop was then trained with features derived from the selected t-score filtering threshold in the inner loop and used to predict the risk group for patients in the left-out test set. This cycle is repeated until every patient slide had a metastasis risk prediction. The number of features for this analysis was restricted by the t-score thresholds. In this feature set, only binary (>50% class probability) and a single broad representation of high (>90%) or low (<10%) metastasis risk scores were included (**Supplementary Table 3**).

### Statistical Analysis

Accuracy, sensitivity/recall, specificity, precision, negative predictive value, balanced accuracy, Matthews Correlation Coefficient, and F1 score were calculated based on confusion matrices. The tissue classification accuracy of the WSI annotation pipeline was evaluated by the Jaccard index. The significance of survival difference was assessed with the log-rank test for Kaplan Meier curves or the Wald chi-square test for Cox regression analysis. *P*-values <0.05 were considered statistically significant. Statistical analyses were performed using the SAS 9.4 software (Cary, NC, USA), Python, and MATLAB R2018b (The MathWorks, MA, USA).

## Results

### Deep Learning WSI Analysis Discriminates Different PanNET Tissues

**Figure 2:**
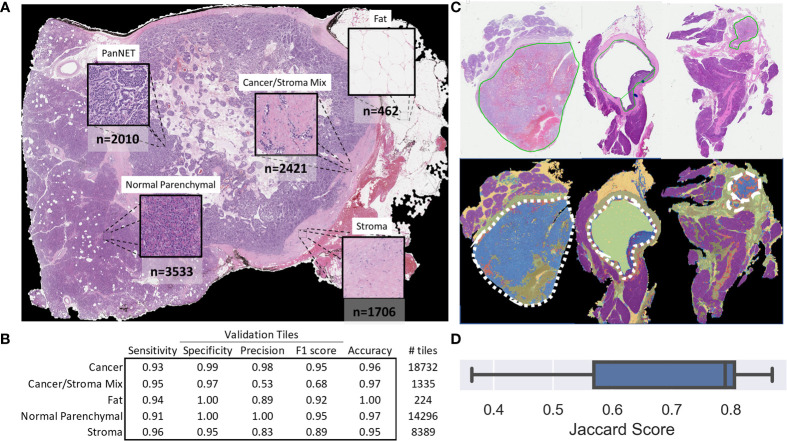
Tile and whole slide performance of the *CNN-based* tissue annotation. **(A)** Examples of the tissue annotation classes and (non-augmented) ground*-*truth tile count*s* used for training. **(B)** The multiclass sensitivity (recall), specificity, precision, F1 score, and accuracy for the validation tiles. **(C)** *Representative* pathologist*-based* annotations (*solid* green line) for cancer regions *and* automated whole-slide annotation (blue: cancer, red: cancer with stroma, purple: normal parenchymal, green: stroma, yellow: fat, major cancer regions outlined with a white dashed line). **(D)** Box plot *showing* the Jaccard score for 11 slides demonstrating the overlap *in CNN-based and pathologist-based* annotation of cancer regions.

A total of 10,132 non-overlapping 150 × 150-pixel, pathologist-annotated, ground-truth regions were extracted (**Figure 2A**) and augmented, providing the 466,072 tiles used to train the annotation CNN. Nine epochs provided an accurate classification of the training data. For the validation data, the CNN provided an overall accuracy of 92.8% and greater than 90% sensitivity and specificity for all annotated classes (**Figure 2B**). Importantly, the CNN provided an F1 score of 0.95 for the validation tiles (n = 42,976) of cancer and normal parenchymal regions. The least precise classification was obtained for cancer/stroma mixed tiles, with an F1 score of 0.68 and a precision value of 0.53, indicating false positive classification, especially toward normal regions (**Supplementary Figure 3** **)**. High concordance was observed between CNN-based and pathologist-based WSI classification (**Figure 2C** and **Supplementary Figure 4**), with a median Jaccard index of 0.79 in cancer regions (**Figure 2D**). Cancer regions were accurately identified, with false-positive areas predominantly in sparse edges/interface areas (less common in the training dataset). These false-positive areas had a low probability (<95%), and, thus, were excluded from subsequent analysis.

### Prognostic Value of the Model

**Figure 3:**
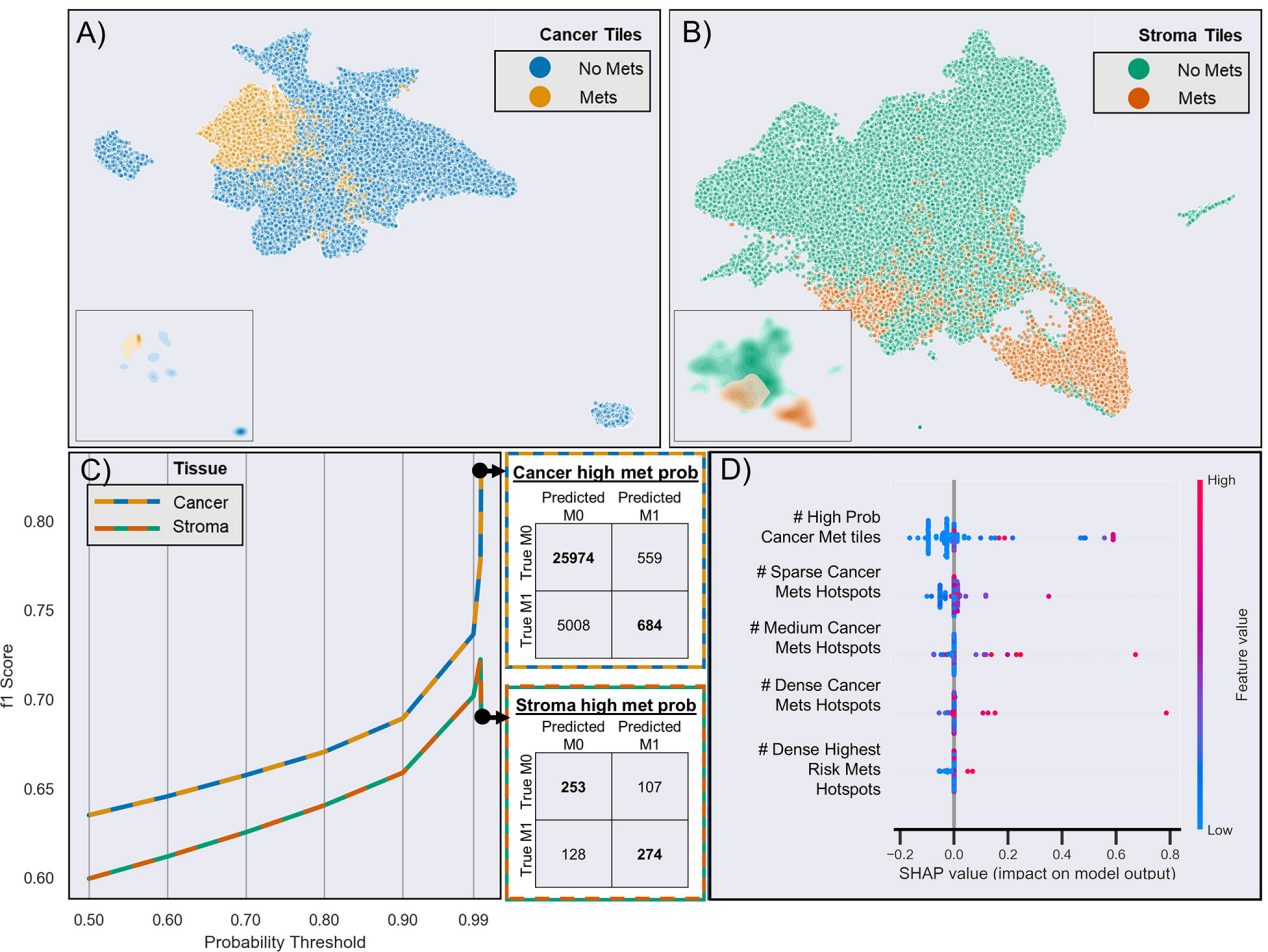
Risk of metastasis in different tiles. **(A)** UMAP clustering of the final CNN pooling layer of the first test-fold cancer tiles across two dimensions. Each point (n = 65,126) represents a different tile, and different colors indicate whether the patient developed metastasis (yellow) or not (blue). The inserts show the density of clustered tiles for each metastasis group. **(B)** UMAP clustering of adjacent stromal tiles (n = 33,555). **(C)** CNN-based prediction performance and CNN probability thresholds for the risk of metastasis in cancer and stroma tiles. The adjacent confusion matrix shows the comparison of the true versus predicted tile labels (M0: no metastasis, M1: metastasis) at the maximum probability (99.99%). **(D)** Selected features based on SHAP analysis of all test samples from the aggregated leave-one-out test sets. The colors represent the feature value, and the SHAP values indicate the importance of each feature in determining a high (>0 SHAP value) or low (<0 SHAP value) risk. For brevity, truncated explanatory titles were used for the feature names: “# High Prob Cancer Met tiles” = “Count Cancer Metastasis Probability Tiles With Prob >=0.9999 and <0.99999”, “# Sparse Cancer Mets Hotspots” = “# of 8 to 9 Met (≥50% probability) Cancer tile clusters,” “# Medium Cancer Mets Hotspots” = “# of 10 to 11 Met (≥50% probability) Cancer tile clusters,” “# Dense Cancer Mets Hotspots” = “# of 16 to 17 Met (≥50% probability) Cancer tile clusters,” “#Dense Highest Risk Mets Hotspots’ = “# of 6 to 7 Met (≥99% probability) Cancer tile clusters.”

CNN-based WSI analysis provided 430,318 cancer and 211,361 stroma tile annotations with a greater than 95% probability and allowed for the creation of a full slide metastasis probability map (**Supplementary Figure 5**). CNN training using these tiles (and the patient’s metastasis status as labels) provided an overall classification test set F1 score of 0.64 and 0.60 for cancer and stroma tiles, respectively. Projection of the final CNN pooling layers from all cross-validated test sets provided a better delineation between cancer tiles and adjacent stroma tiles in patients who developed metastasis than in those that did not (**Figures 3A, B** and **Supplementary Figures 7–9**). Analysis of tiles with higher softmax output probability scores improved the performance for both cancer and stroma regions. That is, classification performance generally increased when analyzing tiles with increasing class probability (going from the default >50% probability output to restricting analysis to those tiles with at least a 99.99% output for either class). For cancer tiles with a maximum analyzed probability score (99.99% softmax output for either the metastasis or non-metastasis class), the F1 score was 0.83, whereas stromal tiles with a 99.9% probability score had an F1 score of 0.72 (**Figure 3C**).

**Figure 4:**
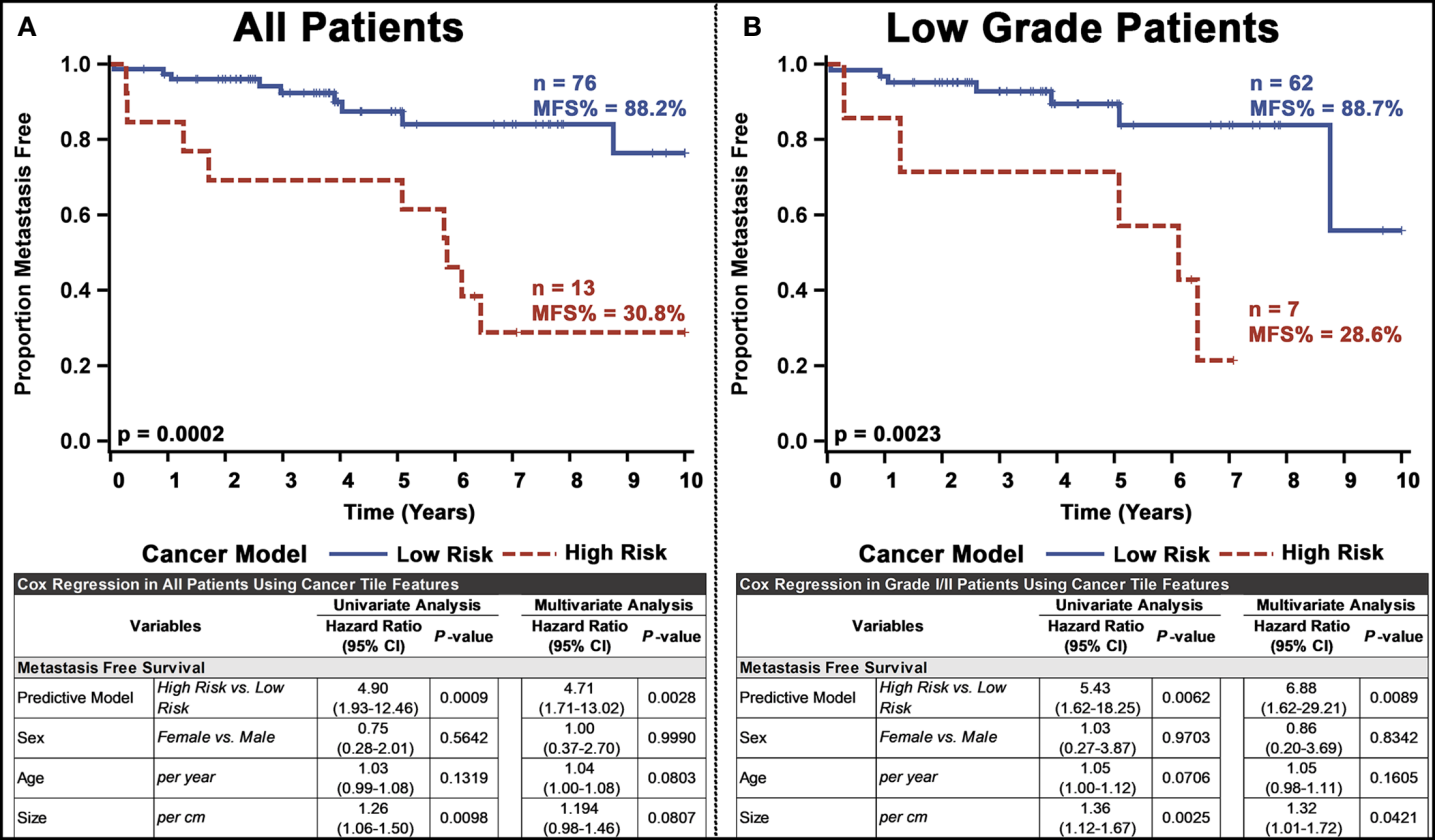
Univariate and multivariate analyses of the PanNET metastasis risk prediction model for: **(A)** all patients and **(B)** Low-grade (I/II) patients. Our ultimate model was the decision tree selected from other models based on the performance of LOOCV on test slides.

Next, we used the whole-slide features to predict the risk of metastasis and found that the use of a decision tree classifier trained using cancer-only features significant above a t-score of 1.2 (when comparing the features values between patients who metastasized versus those who did not in the respective training data) provided the best leave-one-out cross-validation accuracy of 80.77%, properly predicting 84 of 104 slides (**Supplementary Figure 10** and **Supplementary Table 4**. This accuracy was only slightly marginally better than that provided by a model built with all features (slide level accuracy: 78.84, predicting 82 of 104 slides; **Supplementary Figure 11**) or stroma-only features (slide level accuracy: 79.81, predicting 83 of 104 slides; **Supplementary Figure 12**). Furthermore, the model provided concordant predictions for nine of the 14 patients with multiple tissue slides. A high probability of cancer tiles (>0.9%) had the largest impact on the model (**Figure 3D** and **Supplementary Figure 13**). When aggregated from a slide to a patient level, almost 70% of patients determined as high risk developed metastasis within 10 years, whereas this number was only 22% for patients determined as low risk **(** **Figure 4A** and **Supplementary Table 4**). Importantly, the model predicted metastasis-free survival after adjusting for clinical variables, even in low grade (I/II) patients (**Figure 4B**).

Testing the pipelines generalizability through nested cross validation and a reduced, more broadly thresholded, feature set resulted in equally sized high and low risk groups, although with different patient stratifications by our final model. For the full cohort of patients, the pipeline retained significant stratification, even when controlling for other clinopathological variables, although with slightly slower prognostic value (**Supplementary Figure 11A**). Low grade patients, however, did by a small margin lose statistical stratification significance (p = 0.0717) (**Supplementary Figure 14B**).

## Discussion

PanNET is characterized by variable prognosis. Although some patients with metastatic PanNET are treated with surgical resection (27), surgery is only considered a “curative” option for patients with localized disease. In approximately 35% resected PanNET tumors with local disease, metastasis eventually develops (27). Therefore, metastasis remains a concern even in patients with localized, early-stage PanNET (4). Additionally, the relationship between many clinicopathological characteristics and long-term survival after surgery is controversial (1). Although proliferation markers (e.g., Ki67 and mitotic index) have shown a prognostic value, their use suffers from various limitations (28–30). Furthermore, early tumor stages (I/II) have limited prognostic value between themselves in PanNET (31–34). Therefore, the development of novel models to predict outcomes in patients with non-metastatic PanNETs undergoing surgery is of high clinical importance, especially for patients with low-grade tumors.

In this study, we investigated the prognostic value of a novel nested deep learning-based computational pathology model. Deep/machine learning has been demonstrated to outperform humans in various diagnostic tasks, including the interpretation of histologic images of tumors (35), and can identify textural features hidden to the human eye (36). Nested/cascading machine learning approaches have also shown significant discriminatory value (37, 38). Tile-based, segmented/annotated areas have been shown to accurately represent the whole-slide (39–41). Deep learning-based WSI analysis has also been shown to predict patient outcomes (13). Our novel deep learning-based WSI analysis pipeline aggregates metastasis-specific features from relevant tissue areas. Therefore, our model provides useful information on the morphological properties of different tumor regions, which have significant prognostic value in PanNETs (3, 42). Further, our model provides a powerful tool to investigate the surrounding tumor stroma, which has previously been shown to affect PanNET outcomes (43, 44). Automated image analysis pipelines provide a robust characterization of alterations in the tumor stroma (41, 45).

Consistent with previous findings (35), our deep learning-based pipeline provided accurate tissue annotation in PanNETs. Despite some issues with false positive classification of some cancer/stroma tiles, especially toward true normal regions, the overall tissue classification was very good. In over 40,000 validation tiles, the CNN provided an overall annotation accuracy of over 92% for five different tissues. Even in more ambiguous tumor areas, CNN-based WSI annotation was largely in line with the pathologist’s annotation (~80% overlap). Unsurprisingly, CNN-based WSI analysis provided a poor discriminatory power for low-confidence metastasis-associated areas (<50-80% probability), in sharp contrast to its excellent discriminatory ability for high-confidence areas (>99% probability). These findings indicate that stromal and cancer tiles possess morphological features, which are “translated” into a risk of metastasis by the deep learning pipeline. Notably, the model provided higher F1 scores indicating a superior predictive performance when only cancer tiles were analyzed. This cross-validated model identified high-risk patients, who had an over 4.5 times higher risk of developing distant metastasis. Importantly, the model was also able to identify high-risk patients with low-grade PanNET regardless of other clinicopathological variables. Further, using a restrictive nested cross validation and a small feature set to better inspect model generalizability confirmed promising results. Though preliminary, these results demonstrate the ability of our multi-structured deep learning-based model to provide metastasis risk stratification, and potentially facilitate clinical decision making. Additionally, the pipeline described here can help identify patients who might benefit from adjuvant therapy (46) or candidates for clinical trials, as well as enable personalized treatment (47).

Despite these encouraging findings, the study has limitations. First, our cohort was from a single institution, and given the relatively low incidence of PanNETs and high incidence of distant metastasis (48), our findings require comprehensive external validation. Second, it is important to increase the size of training data to better fit the prognostic model. This is also vital to increase the model sensitivity and capture a higher proportion of metastatic patients. When controlling through a nested cross-validation approach, low-grade patients had a significant stratification group suggesting additional work toward developing a more generalizable model. Furthermore, our pipeline did not consider important clinicopathological variables, such as tumor stage components (outside of tumor size). Although the CNN provided satisfactory discrimination of the different tissues in the validation sets, further investigation is required to determine the influence of false positives and true negatives on whole-slide annotation. Metastasis association scores we calculated separately for cancer and adjacent stromal tiles. However, CNNs could be trained to identify metastatic signatures in other tissues, such as the normal parenchymal (49). Finally, although our analysis involved investigating a robust selection of algorithms, the implementation of additional methods, such as survival trees, could further improve model interpretability. More extensive CNN training and implementation is justified. More complex, state of the art networks could further improve model performance whereas simpler models could potentially retain classification performance with significantly improved training speed (50).

In conclusion, our findings provide initial evidence that our novel, multiclassification, deep learning pipeline can predict the risk of metastasis in PanNET patients, by using H&E sections of surgically resected tissue. Our results also suggest that prognostic morphological patterns exist among PanNETs, both within the tumor as well as the adjacent stromal regions. Future studies, in a larger cohort with available outcome and treatment data, are warranted to further investigate the potential value of such morphological markers in guiding clinical decision making.

## Data Availability Statement

The original contributions presented in the study are included in the Supplementary Materials. Further inquiries can be directed to the corresponding authors.

## Ethics Statement

Study was approved through an institutional IRB (IRB00083114: Emory Neuroendocrine Neoplasms) and a waiver of consent.

## Author Contributions

SK and MR: conception and design. SK and AG: data analysis, image analysis, statistical analysis, and manuscript writing. YX, SB, SP, and RG: collection of data and materials. YX and MR: case screening and annotation. All authors: data interpretation, critical manuscript review and revision, and approval of the final manuscript. All authors contributed to the article and approved the submitted version.

## Funding

This study was supported by a grant from the National Cancer Institute (U01 CA179671) to RA.

## Conflict of Interest

The authors declare that the research was conducted in the absence of any commercial or financial relationships that could be construed as a potential conflict of interest.

## Supplementary Material

Supplementary Presentation 1 The supplementary figures and tables.

Supplementary Data sheet 1 Montages of tiles found to be a high/low metastasis risk and with/without future metastasis.

Supplementary Data Sheet 2 The algorithms and scripts used throughout the paper.

Supplementary Data Sheet 3 The trained metastasis classification CNN for cancer tiles.

Supplementary Data Sheet 4 The trained tile annotation classifier.

Supplementary Data Sheet 5 The trained metastasis classification CNN for stroma tiles.

## References

[1] Ehehalt F Saeger HD Schmidt CM Grützmann R. Neuroendocrine tumors of the pancreas. Oncologist (2009) 14(5):456–67. 10.1634/theoncologist.2008-0259 19411317

[2] Leung HH Chan AW. Updates of pancreatic neuroendocrine neoplasm in the 2017 World Health Organization classification. Surg Pract (2019) 23(2):42–7. 10.1111/1744-1633.12353

[3] Basturk O Yang Z Tang LH Hruban RH Adsay NV McCall CM. The high grade (WHO G3) pancreatic neuroendocrine tumor category is morphologically and biologically heterogeneous and includes both well differentiated and poorly differentiated neoplasms. Am J Surg Pathol (2015) 39(5):683. 10.1097/PAS.0000000000000408 25723112 PMC4398606

[4] Reid MD Balci S Saka B Adsay NV. Neuroendocrine tumors of the pancreas: current concepts and controversies. Endocr Pathol (2014) 25(1):65–79. 10.1007/s12022-013-9295-2 24430597

[5] Viúdez A Carvalho FL Maleki Z Zahurak M Laheru D Stark A. A new immunohistochemistry prognostic score (IPS) for recurrence and survival in resected pancreatic neuroendocrine tumors (PanNET). Oncotarget (2016) 7(18):24950. 10.18632/oncotarget.7436 26894863 PMC5041882

[6] Gao H Liu L Wang W Xu H Jin K Wu C. Novel recurrence risk stratification of resected pancreatic neuroendocrine tumor. Cancer Lett (2018) 412:188–93. 10.1016/j.canlet.2017.10.036 29107104

[7] Tang LH Gonen M Hedvat C Modlin IM Klimstra DS. Objective quantification of the Ki67 proliferative index in neuroendocrine tumors of the gastroenteropancreatic system: a comparison of digital image analysis with manual methods. Am J Surg Pathol (2012) 36(12):1761–70. 10.1097/PAS.0b013e318263207c 23026928

[8] Rindi G Falconi M Klersy C Albarello L Boninsegna L Buchler M. TNM staging of neoplasms of the endocrine pancreas: results from a large international cohort study. J Natl Cancer Inst (2012) 104(10):764–77. 10.1093/jnci/djs208 22525418

[9] Salaria SN Shi C. Pancreatic neuroendocrine tumors. Surg Pathol Clinics (2016) 9(4):595–617. 10.1016/j.path.2016.05.006 27926362

[10] Tang LH Untch BR Reidy DL O’Reilly E Dhall D Jih L. Well-differentiated neuroendocrine tumors with a morphologically apparent high-grade component: a pathway distinct from poorly differentiated neuroendocrine carcinomas. Clin Cancer Res (2016) 22(4):1011–7. 10.1158/1078-0432.CCR-15-0548 PMC4988130 26482044

[11] Xue Y Reid MD Pehlivanoglu B Obeng RC Jiang H Memis B. Morphologic Variants of Pancreatic Neuroendocrine Tumors: Clinicopathologic Analysis and Prognostic Stratification. Endocr Pathol (2020) 31(3):239–53. 10.1007/s12022-020-09628-z 32488621

[12] Gertych A Swiderska-Chadaj Z Ma Z Ing N Markiewicz T Cierniak S. Convolutional neural networks can accurately distinguish four histologic growth patterns of lung adenocarcinoma in digital slides. Sci Rep (2019) 9(1):1483. 10.1038/s41598-018-37638-9 30728398 PMC6365499

[13] Mobadersany P Yousefi S Amgad M Gutman DA Barnholtz-Sloan JS Vega JEV. Predicting cancer outcomes from histology and genomics using convolutional networks. Proc Natl Acad Sci (2018) 115(13):E2970–E9. 10.1073/pnas.1717139115 PMC5879673 29531073

[14] Osamura R Grossman A Korbonits M Kovacs K Lopes M Matsuno A. WHO Classification of Tumours of Endocrine Organs. France: IARC Lyon (2017).

[15] Szegedy C Liu W Jia Y Sermanet P Reed S Anguelov D. eds. Going deeper with convolutions. In: Proceedings of the IEEE conference on computer vision and pattern recognition. (2015).

[16] Mathworks I. MATLAB and computer vision release 2020b. Massachusetts, United States: Natick (2020).

[17] Reinhard E Adhikhmin M Gooch B Shirley P. Color transfer between images. IEEE Comput Graphics Appl (2001) 21(5):34–41. 10.1109/38.946629

[18] Janowczyk A Basavanhally A Madabhushi A. Stain normalization using sparse autoencoders (StaNoSA): Application to digital pathology. Comput Med Imaging Graphics (2017) 57:50–61. 10.1016/j.compmedimag.2016.05.003 PMC5112159 27373749

[19] Araújo T Aresta G Castro E Rouco J Aguiar P Eloy C. Classification of breast cancer histology images using convolutional neural networks. PloS One (2017) 12(6):e0177544. 10.1371/journal.pone.0177544 28570557 PMC5453426

[20] McInnes L Healy J Melville J. Umap: Uniform manifold approximation and projection for dimension reduction. arXiv preprint arXiv:180203426 (2018). 10.21105/joss.00861

[21] Connor Meehan SM Moore W. Uniform Manifold Approximation and Projection (UMAP). MATLAB Central File Exchange (2020). Available at: https://www.mathworks.com/matlabcentral/fileexchange/71902.

[22] Buuren SV Groothuis-Oudshoorn K. mice: Multivariate imputation by chained equations in R. J Stat Softw (2010) 45:1–68. 10.18637/jss.v045.i03

[23] Guyon I Elisseeff A. An introduction to variable and feature selection. J Mach Learn Res (2003) 3(Mar):1157–82. 10.5555/944919.944968

[24] Lundberg SM Lee S-I eds. A unified approach to interpreting model predictions. arXiv preprint arXiv:170507874 (2017).

[25] Lundberg SM Erion G Chen H DeGrave A Prutkin JM Nair B. From local explanations to global understanding with explainable AI for trees. Nat Mach Intell (2020) 2(1):2522–5839. 10.1038/s42256-019-0138-9 PMC7326367 32607472

[26] Lundberg SM Nair B Vavilala MS Horibe M Eisses MJ Adams T. Explainable machine-learning predictions for the prevention of hypoxaemia during surgery. Nat Biomed Eng (2018) 2(10):749–60. 10.1038/s41551-018-0304-0 PMC6467492 31001455

[27] Hill JS McPhee JT McDade TP Zhou Z Sullivan ME Whalen GF. Pancreatic neuroendocrine tumors: the impact of surgical resection on survival. Cancer (2009) 115(4):741–51. 10.1002/cncr.24065 19130464

[28] McCall CM Shi C Cornish TC Klimstra DS Tang LH Basturk O. Grading of well-differentiated pancreatic neuroendocrine tumors is improved by the inclusion of both Ki67 proliferative index and mitotic rate. Am J Surg Pathol (2013) 37(11):1671. 10.1097/PAS.0000000000000089 24121170 PMC3891823

[29] Singhi AD Klimstra DS. Well-differentiated pancreatic neuroendocrine tumours (Pan NET s) and poorly differentiated pancreatic neuroendocrine carcinomas (Pan NEC s): concepts, issues and a practical diagnostic approach to high-grade (G3) cases. Histopathology (2018) 72(1):168–77. 10.1111/his.13408 29239037

[30] Jamali M Chetty R. Predicting prognosis in gastroentero-pancreatic neuroendocrine tumors: an overview and the value of Ki-67 immunostaining. Endocr Pathol (2008) 19(4):282. 10.1007/s12022-008-9044-0 18931958

[31] Ekeblad S Skogseid B Dunder K Öberg K Eriksson B. Prognostic Factors and Survival in 324 Patients with Pancreatic Endocrine Tumor Treated at a Single Institution. Clin Cancer Res (2008) 14(23):7798–803. 10.1158/1078-0432.Ccr-08-0734 19047107

[32] Fischer L Kleeff J Esposito I Hinz U Zimmermann A Friess H. Clinical outcome and long-term survival in 118 consecutive patients with neuroendocrine tumours of the pancreas. Br J Surg: Incorporating Eur J Surg Swiss Surg (2008) 95(5):627–35. 10.1002/bjs.6051 18306152

[33] La Rosa S Klersy C Uccella S Dainese L Albarello L Sonzogni A. Improved histologic and clinicopathologic criteria for prognostic evaluation of pancreatic endocrine tumors. Hum Pathol (2009) 40(1):30–40. 10.1016/j.humpath.2008.06.005 18715612

[34] Pape U-F Jann H Müller-Nordhorn J Bockelbrink A Berndt U Willich SN. Prognostic relevance of a novel TNM classification system for upper gastroenteropancreatic neuroendocrine tumors. Cancer (2008) 113(2):256–65. 10.1002/cncr.23549 18506737

[35] Srinidhi CL Ciga O Martel AL. Deep neural network models for computational histopathology: A survey. Med Image Anal (2020) 101813. 33049577 10.1016/j.media.2020.101813 PMC7725956

[36] Coudray N Ocampo PS Sakellaropoulos T Narula N Snuderl M Fenyö D. Classification and mutation prediction from non–small cell lung cancer histopathology images using deep learning. Nat Med (2018) 24(10):1559–67. 10.1038/s41591-018-0177-5 PMC9847512 30224757

[37] Doyle S Feldman MD Shih N Tomaszewski J Madabhushi A. Cascaded discrimination of normal, abnormal, and confounder classes in histopathology: Gleason grading of prostate cancer. BMC Bioinformatics (2012) 13(1):282. 10.1186/1471-2105-13-282 23110677 PMC3563463

[38] Wang H Viswanath S Madabhushi A. Discriminative scale learning (DiScrn): Applications to prostate cancer detection from MRI and needle biopsies. Sci Rep (2017) 7(1):1–13. 10.1038/s41598-017-12569-z 28959011 PMC5620056

[39] AbdulJabbar K Raza SEA Rosenthal R Jamal-Hanjani M Veeriah S Akarca A. Geospatial immune variability illuminates differential evolution of lung adenocarcinoma. Nat Med (2020) 26:1–9. 10.1038/s41591-020-0900-x 32461698 PMC7610840

[40] Klimov S Miligy IM Gertych A Jiang Y Toss MS Rida P. A whole slide image-based machine learning approach to predict ductal carcinoma in situ (DCIS) recurrence risk. Breast Cancer Res (2019) 21(1):83. 10.1186/s13058-019-1165-5 31358020 PMC6664779

[41] Kather JN Krisam J Charoentong P Luedde T Herpel E Weis C-A. Predicting survival from colorectal cancer histology slides using deep learning: A retrospective multicenter study. PloS Med (2019) 16(1):e1002730. 10.1371/journal.pmed.1002730 30677016 PMC6345440

[42] Xue Y Reid MD Pehlivanoglu B Obeng RC Jiang H Memis B. Morphologic Variants of Pancreatic Neuroendocrine Tumors: Clinicopathologic Analysis and Prognostic Stratification. Endocr Pathol (2020) 31:239–53. 10.1007/s12022-020-09628-z 32488621

[43] Cai L Michelakos T Deshpande V Arora KS Yamada T Ting DT. Role of tumor-associated macrophages in the clinical course of pancreatic neuroendocrine tumors (PanNETs). Clin Cancer Res (2019) 25(8):2644–55. 10.1158/1078-0432.CCR-18-1401 PMC6582654 30670493

[44] Johnson A Wright JP Zhao Z Komaya T Parikh A Merchant N. Cadherin 17 is frequently expressed by ‘sclerosing variant’pancreatic neuroendocrine tumour. Histopathology (2015) 66(2):225–33. 10.1111/his.12535 PMC4302003 25307987

[45] Beck AH Sangoi AR Leung S Marinelli RJ Nielsen TO Van De Vijver MJ. Systematic analysis of breast cancer morphology uncovers stromal features associated with survival. Sci Trans Med (2011) 3(108):108ra13–ra13. 10.1126/scitranslmed.3002564 22072638

[46] Akirov A Larouche V Alshehri S Asa SL Ezzat S. Treatment options for pancreatic neuroendocrine tumors. Cancers (2019) 11(6):828. 10.3390/cancers11060828 PMC6628351 31207914

[47] Kelgiorgi D Dervenis C. Pancreatic neuroendocrine tumors: the basics, the gray zone, and the target. F1000Research (2017) 6:663–. 10.12688/f1000research.10188.1 PMC5428491 28529726

[48] Yao JC Hassan M Phan A Dagohoy C Leary C Mares JE. One hundred years after “carcinoid”: epidemiology of and prognostic factors for neuroendocrine tumors in 35,825 cases in the United States. J Clin Oncol (2008) 26(18):3063–72. 10.1200/JCO.2007.15.4377 18565894

[49] Aran D Camarda R Odegaard J Paik H Oskotsky B Krings G. Comprehensive analysis of normal adjacent to tumor transcriptomes. Nat Commun (2017) 8(1):1–14. 10.1038/s41467-017-01027-z 29057876 PMC5651823

[50] Kather JN Heij LR Grabsch HI Loeffler C Echle A Muti HS. Pan-cancer image-based detection of clinically actionable genetic alterations. Nat Cancer (2020) 1(8):789–99. 10.1038/s43018-020-0087-6 PMC7610412 33763651

