# Supplementary material for: Predicting Metastasis Risk in Pancreatic Neuroendocrine Tumors Using Deep Learning Image Analysis
Source: Front Oncol. 2021 Feb 25;10:593211. doi: 10.3389/fonc.2020.593211 (PMC7946991; doi:10.3389/fonc.2020.593211)
Supplement: Supplementary Presentation 1 — The supplementary figures and tables. [file Presentation_1.pdf]

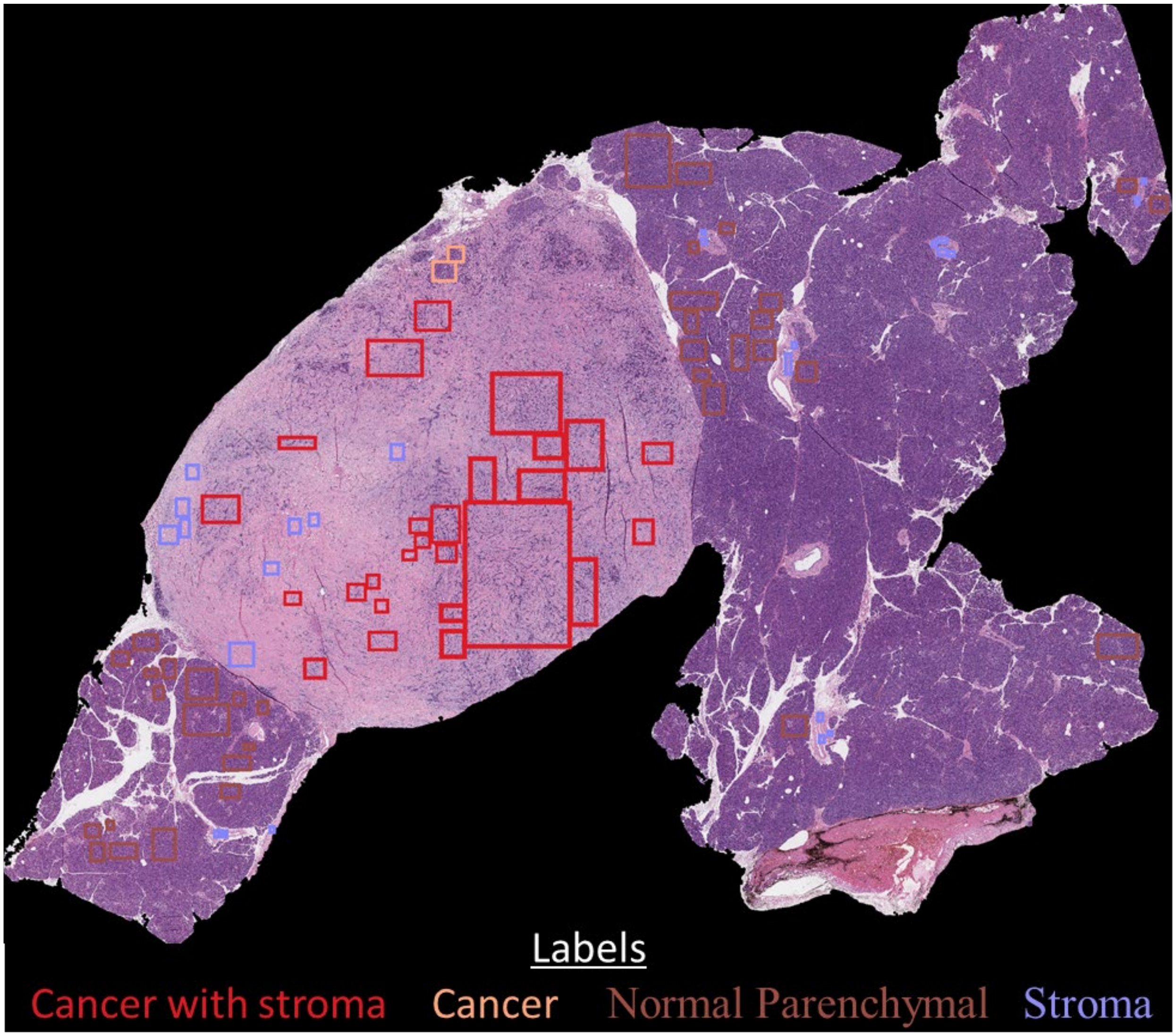

Supplementary Figure 1: Example of a labeled, annotated, slide from which ground truth regions are taken from within overlaid bounding boxes. Fat annotation not present.

Supplementary Table 1: Whole slide feature set

Feature #	Feature
1	Full Cancer Metastasis Probability Histogram Mean
2	Full Cancer Metastasis Probability Histogram Std
3	Full Cancer Metastasis Probability Histogram Skew
4	Full Cancer Metastasis Probability Histogram Kurtosis
5	High Prob (>=0.9) Cancer Metastasis Probability Histogram Mean
6	High Prob (>=0.9) Cancer Metastasis Probability Histogram Std
7	High Prob (>=0.9) Cancer Metastasis Probability Histogram Skew
8	High Prob (>=0.9) Cancer Metastasis Probability Histogram Kurtosis
9	Count Cancer Metastasis Probability Tiles With Prob >=0.9 and <0.95
10	Count Cancer Metastasis Probability Tiles With Prob >=0.95 and <0.99
11	Count Cancer Metastasis Probability Tiles With Prob >=0.99 and <0.999
12	Count Cancer Metastasis Probability Tiles With Prob >=0.999 and <0.9999
13	Count Cancer Metastasis Probability Tiles With Prob >=0.9999 and <0.99999
14	Count Cancer Metastasis Probability Tiles With Prob >=0.99999
15	Proportion Cancer Metastasis Probability Tiles With Prob >=0.9 and <0.95
16	Proportion Cancer Metastasis Probability Tiles With Prob >=0.95 and <0.99
17	Proportion Cancer Metastasis Probability Tiles With Prob >=0.99 and <0.999
18	Proportion Cancer Metastasis Probability Tiles With Prob >=0.999 and <0.9999
19	Proportion Cancer Metastasis Probability Tiles With Prob >=0.9999 and <0.99999
20	Proportion Cancer Metastasis Probability Tiles With Prob >=0.99999
21	Low Prob (<=0.1) Cancer Metastasis Probability Histogram Mean
22	Low Prob (<=0.1) Cancer Metastasis Probability Histogram Std
23	Low Prob (<=0.1) Cancer Metastasis Probability Histogram Skew
24	Low Prob (<=0.1) Cancer Metastasis Probability Histogram Kurtosis
25	Count Cancer Metastasis Probability Tiles With Prob <=0.1
26	Count Cancer Metastasis Probability Tiles With Prob <= 0.05
27	Count Cancer Metastasis Probability Tiles With Prob <= 0.01
28	Count Cancer Metastasis Probability Tiles With Prob <= 0.001
29	Count Cancer Metastasis Probability Tiles With Prob <= 0.0001
30	Count Cancer Metastasis Probability Tiles With Prob <= 0.00001
31	Proportion Cancer Metastasis Probability Tiles With Prob <=0.1
32	Proportion Cancer Metastasis Probability Tiles With Prob <= 0.05
33	Proportion Cancer Metastasis Probability Tiles With Prob <= 0.01
34	Proportion Cancer Metastasis Probability Tiles With Prob <= 0.001
35	Proportion Cancer Metastasis Probability Tiles With Prob <= 0.0001
36	Proportion Cancer Metastasis Probability Tiles With Prob <= 0.00001
37	Cancer Metastasis Probability Distribution Entropy
38	Full Nearby Stroma Metastasis Probability Histogram Mean
39	Full Nearby Stroma Metastasis Probability Histogram Std
40	Full Nearby Stroma Metastasis Probability Histogram Skew
41	Full Nearby Stroma Metastasis Probability Histogram Kurtosis
42	High Prob (>=0.9) Nearby Stroma Metastasis Probability Histogram Mean

43	High Prob (>=0.9) Nearby Stroma Metastasis Probability Histogram Std
44	High Prob (>=0.9) Nearby Stroma Metastasis Probability Histogram Skew
45	High Prob (>=0.9) Nearby Stroma Metastasis Probability Histogram Kurtosis
46	Count Nearby Stroma Metastasis Probability Tiles With Prob >=0.9 and <0.95
47	Count Nearby Stroma Metastasis Probability Tiles With Prob >=0.95 and <0.99
48	Count Nearby Stroma Metastasis Probability Tiles With Prob >=0.99 and <0.999
49	Count Nearby Stroma Metastasis Probability Tiles With Prob >=0.999 and <0.9999
50	Count Nearby Stroma Metastasis Probability Tiles With Prob >=0.9999 and <0.99999
51	Count Nearby Stroma Metastasis Probability Tiles With Prob >=0.99999
52	Proportion Nearby Stroma Metastasis Probability Tiles With Prob >=0.9 and <0.95
53	Proportion Nearby Stroma Metastasis Probability Tiles With Prob >=0.95 and <0.99
54	Proportion Nearby Stroma Metastasis Probability Tiles With Prob >=0.99 and <0.999
55	Proportion Nearby Stroma Metastasis Probability Tiles With Prob >=0.999 and <0.9999
56	Proportion Nearby Stroma Metastasis Probability Tiles With Prob >=0.9999 and <0.99999
57	Proportion Nearby Stroma Metastasis Probability Tiles With Prob >=0.99999
58	Low Prob (<=0.1) Nearby Stroma Metastasis Probability Histogram Mean
59	Low Prob (<=0.1) Nearby Stroma Metastasis Probability Histogram Std
60	Low Prob (<=0.1) Nearby Stroma Metastasis Probability Histogram Skew
61	Low Prob (<=0.1) Nearby Stroma Metastasis Probability Histogram Kurtosis
62	Count Nearby Stroma Metastasis Probability Tiles With Prob <=0.1
63	Count Nearby Stroma Metastasis Probability Tiles With Prob <= 0.05
64	Count Nearby Stroma Metastasis Probability Tiles With Prob <= 0.01
65	Count Nearby Stroma Metastasis Probability Tiles With Prob <= 0.001
66	Count Nearby Stroma Metastasis Probability Tiles With Prob <= 0.0001
67	Count Nearby Stroma Metastasis Probability Tiles With Prob <= 0.00001
68	Proportion Nearby Stroma Metastasis Probability Tiles With Prob <=0.1
69	Proportion Nearby Stroma Metastasis Probability Tiles With Prob <= 0.05
70	Proportion Nearby Stroma Metastasis Probability Tiles With Prob <= 0.01
71	Proportion Nearby Stroma Metastasis Probability Tiles With Prob <= 0.001
72	Proportion Nearby Stroma Metastasis Probability Tiles With Prob <= 0.0001
73	Proportion Nearby Stroma Metastasis Probability Tiles With Prob <= 0.00001
74	Nearby Stroma Metastasis Probability Distribution Entropy
75	Nearby Stroma count prob (0-9%)
76	Nearby Stroma count prob (10-19%)
77	Nearby Stroma count prob (20-29%)
78	Nearby Stroma count prob (30-39%)
79	Nearby Stroma count prob (40-49%)
80	Nearby Stroma count prob (50-59%)
81	Nearby Stroma count prob (60-69%)
82	Nearby Stroma count prob (70-79%)
83	Nearby Stroma count prob (80-89%)
84	Nearby Stroma count prob (>=90%)
85	Cancer count prob (0-9%)
86	Cancer count prob (10-19%)
87	Cancer count prob (20-29%)

88	Cancer count prob (30-39%)
89	Cancer count prob (40-49%)
90	Cancer count prob (50-59%)
91	Cancer count prob (60-69%)
92	Cancer count prob (70-79%)
93	Cancer count prob (80-89%)
94	Cancer count prob (>=90%)
95	# of 0 to 2 Met (>=50% probability) Cancer tile clusters
96	# of 2 to 3 Met (>=50% probability) Cancer tile clusters
97	# of 4 to 5 Met (>=50% probability) Cancer tile clusters
98	# of 6 to 7 Met (>=50% probability) Cancer tile clusters
99	# of 8 to 9 Met (>=50% probability) Cancer tile clusters
100	# of 10 to 11 Met (>=50% probability) Cancer tile clusters
101	# of 12 to 13 Met (>=50% probability) Cancer tile clusters
102	# of 14 to 15 Met (>=50% probability) Cancer tile clusters
103	# of 16 to 17 Met (>=50% probability) Cancer tile clusters
104	# of 18 to 19 Met (>=50% probability) Cancer tile clusters
105	# of 20 to 21 Met (>=50% probability) Cancer tile clusters
106	# of 22 to 23 Met (>=50% probability) Cancer tile clusters
107	# of 24 to 25 Met (>=50% probability) Cancer tile clusters
108	# of 26 to 27 Met (>=50% probability) Cancer tile clusters
109	# of 28 to 30+ Met (>=50% probability) Cancer tile clusters
110	# of 30+ Met (>=50% probability) Cancer tile clusters
111	# of 0 to 2 Met (>=90% probability) Cancer tile clusters
112	# of 2 to 3 Met (>=90% probability) Cancer tile clusters
113	# of 4 to 5 Met (>=90% probability) Cancer tile clusters
114	# of 6 to 7 Met (>=90% probability) Cancer tile clusters
115	# of 8 to 10+ Met (>=90% probability) Cancer tile clusters
116	# of 10+ Met (>=90% probability) Cancer tile clusters
117	# of 0 to 2 Met (>=99% probability) Cancer tile clusters
118	# of 2 to 3 Met (>=99% probability) Cancer tile clusters
119	# of 4 to 5 Met (>=99% probability) Cancer tile clusters
120	# of 6 to 7 Met (>=99% probability) Cancer tile clusters
121	# of 8 to 10+ Met (>=99% probability) Cancer tile clusters
122	# of 10+ Met (>=99% probability) Cancer tile clusters
123	# of 0 to 2 Met (>=50% probability) Nearby Stroma tile clusters
124	# of 2 to 3 Met (>=50% probability) Nearby Stroma tile clusters
125	# of 4 to 5 Met (>=50% probability) Nearby Stroma tile clusters
126	# of 6 to 7 Met (>=50% probability) Nearby Stroma tile clusters
127	# of 8 to 9 Met (>=50% probability) Nearby Stroma tile clusters
128	# of 10 to 11 Met (>=50% probability) Nearby Stroma tile clusters
129	# of 12 to 13 Met (>=50% probability) Nearby Stroma tile clusters
130	# of 14 to 15 Met (>=50% probability) Nearby Stroma tile clusters
131	# of 16 to 17 Met (>=50% probability) Nearby Stroma tile clusters
132	# of 18 to 19 Met (>=50% probability) Nearby Stroma tile clusters

133	# of 20 to 21 Met (>=50% probability) Nearby Stroma tile clusters
134	# of 22 to 23 Met (>=50% probability) Nearby Stroma tile clusters
135	# of 24 to 25 Met (>=50% probability) Nearby Stroma tile clusters
136	# of 26 to 27 Met (>=50% probability) Nearby Stroma tile clusters
137	# of 28 to 30+ Met (>=50% probability) Nearby Stroma tile clusters
138	# of 30+ Met (>=50% probability) Nearby Stroma tile clusters
139	# of 0 to 2 Met (>=90% probability) Nearby Stroma tile clusters
140	# of 2 to 3 Met (>=90% probability) Nearby Stroma tile clusters
141	# of 4 to 5 Met (>=90% probability) Nearby Stroma tile clusters
142	# of 6 to 7 Met (>=90% probability) Nearby Stroma tile clusters
143	# of 8 to 10+ Met (>=90% probability) Nearby Stroma tile clusters
144	# of 10+ Met (>=90% probability) Nearby Stroma tile clusters
145	# of 0 to 2 Met (>=99% probability) Nearby Stroma tile clusters
146	# of 2 to 3 Met (>=99% probability) Nearby Stroma tile clusters
147	# of 4 to 5 Met (>=99% probability) Nearby Stroma tile clusters
148	# of 6 to 7 Met (>=99% probability) Nearby Stroma tile clusters
149	# of 8 to 10+ Met (>=99% probability) Nearby Stroma tile clusters
150	# of 10+ Met (>=99% probability) Nearby Stroma tile clusters

Supplementary Table 2: List of all machine learning models used as part of the 'zoo'. KNN: K-Nearest Neighbor, SVM: Support Vector Machine. *: changes to the default MATLAB classifier, **: MATLAB function used to train the respective models.

	Machine Learning Models	HyperParameters*	Function**
1 -	Fine decision tree	MaxNumSplits: 100	fitctree
2 -	Medium decision tree	MaxNumSplits: 200	fitctree
3 -	Coarse decision tree	MaxNumSplits: 4	fitctree
4 -	Fine KNN	NumNeighbors: 1, Standardize: Yes	fitcknn
5 -	Medium KNN	NumNeighbors: 10, Standardize: Yes	fitcknn
6 -	Coarse KNN	NumNeighbors: 100, Standardize: Yes	fitcknn
7 -	Cosine KNN	NumNeighbors: 10, Distance: cosine	fitcknn
8 -	Cubic KNN	NumNeighbors: 10, Distance: minkowski	fitcknn
9 -	Weighten KNN	NumNeighbors: 10, Distance: squaredinverse	fitcknn
10 -	Linear SVM	KernelFunction: linear, BoxConstraint: 1, Standardize: Yes, KernelScale: auto	fitcsvm
11 -	Quadratic SVM	KernelFunction: polynomial, PolynomialOrder: 2, BoxConstraint: 1, Standardize: Yes, KernelScale: auto	fitcsvm
12 -	Cubic SVM	KernelFunction: polynomial, PolynomialOrder: 3, BoxConstraint: 1, Standardize: Yes, KernelScale: auto	fitcsvm
13 -	Fine guassian SVM	KernelFunction: gaussian, BoxConstraint: 1, Standardize: Yes, KernelScale: 1.2	fitcsvm
14 -	Medium guassian SVM	KernelFunction: gaussian, BoxConstraint: 1, Standardize: Yes, KernelScale: 4.8	fitcsvm
15 -	Coarse guassian SVM	KernelFunction: gaussian, BoxConstraint: 1, Standardize: Yes, KernelScale: 19	fitcsvm
16 -	Ensemble boosted trees	Method: AdaBoostM1, MaxNumSplits: 20, LearnRate: 0.1	fitcensemble
17 -	Ensemble bagged trees	Method: Bag, MaxNumSplits: NumObservations-1	fitcensemble
18 -	RUSboost trees	Method: RUSBoost, MaxNumSplits: 20, LearnRate: 0.1	fitcensemble

Supplementary Table 3: Generalized whole slide feature list.

Feature #	Feature
1	Full Cancer Metastasis Probability Histogram Mean
2	Full Cancer Metastasis Probability Histogram Std

3	Full Cancer Metastasis Probability Histogram Skew
4	Full Cancer Metastasis Probability Histogram Kurtosis
5	Binary (>.5) Metastasis Probability Histogram Mean
6	Binary (>.5) Metastasis Probability Histogram Std
7	Binary (>.5) Metastasis Probability Histogram Skew
8	Binary (>.5) Metastasis Probability Histogram Kurtosis
9	Binary (<.5) Metastasis Probability Histogram Mean
10	Binary (<.5) Metastasis Probability Histogram Std
11	Binary (<.5) Metastasis Probability Histogram Skew
12	Binary (<.5) Metastasis Probability Histogram Kurtosis
13	High Prob (>=0.9) Cancer Metastasis Probability Histogram Mean
14	High Prob (>=0.9) Cancer Metastasis Probability Histogram Std
15	High Prob (>=0.9) Cancer Metastasis Probability Histogram Skew
16	High Prob (>=0.9) Cancer Metastasis Probability Histogram Kurtosis
17	Count Cancer Metastasis Probability Tiles With Prob >0.5
18	Count Cancer Metastasis Probability Tiles With Prob >=0.9
19	Proportion Cancer Metastasis Probability Tiles With Prob >0.5
20	Proportion Cancer Metastasis Probability Tiles With Prob >=0.9
21	Low Prob (<=0.1) Cancer Metastasis Probability Histogram Mean
22	Low Prob (<=0.1) Cancer Metastasis Probability Histogram Std
23	Low Prob (<=0.1) Cancer Metastasis Probability Histogram Skew
24	Low Prob (<=0.1) Cancer Metastasis Probability Histogram Kurtosis
25	Count Cancer Metastasis Probability Tiles With Prob <0.5
26	Count Cancer Metastasis Probability Tiles With Prob <=0.1
27	Proportion Cancer Metastasis Probability Tiles With Prob <0.5
28	Proportion Cancer Metastasis Probability Tiles With Prob <=0.1
29	Cancer Metastasis Probability Distribution Entrophy
30	# of Met (>=50% probability) Cancer tile clusters
31	# of No Met (>=50% probability) Cancer tile clusters
32	# of 1 to 10 Met (>=50% probability) Cancer tile clusters
33	# of 11 to 20 Met (>=50% probability) Cancer tile clusters
34	# of 21 to 30 Met (>=50% probability) Cancer tile clusters
35	# of >30 Met (>=50% probability) Cancer tile clusters
36	# of No Met (>=50% probability) Cancer tile clusters
37	# of 1 to 5 Met (>=90% probability) Cancer tile clusters
38	# of 6 to 10 Met (>=90% probability) Cancer tile clusters
39	# of >10 Met (>=90% probability) Cancer tile clusters
40	Proportion of No Met (>=50% probability) Cancer tile clusters
41	Proportion of 1 to 10 Met (>=50% probability) Cancer tile clusters
42	Proportion of 11 to 20 Met (>=50% probability) Cancer tile clusters
43	Proportion of 21 to 30 Met (>=50% probability) Cancer tile clusters
44	Proportion of >30 Met (>=50% probability) Cancer tile clusters
45	Proportion of No Met (>=50% probability) Cancer tile clusters

46	Proportion of 1 to 5 Met (>=90% probability) Cancer tile clusters
47	Proportion of 6 to 10 Met (>=90% probability) Cancer tile clusters
48	Proportion of >10 Met (>=90% probability) Cancer tile clusters
49	Full Stroma Metastasis Probability Histogram Mean
50	Full Stroma Metastasis Probability Histogram Std
51	Full Stroma Metastasis Probability Histogram Skew
52	Full Stroma Metastasis Probability Histogram Kurtosis
53	Binary (>.5) Metastasis Probability Histogram Mean
54	Binary (>.5) Metastasis Probability Histogram Std
55	Binary (>.5) Metastasis Probability Histogram Skew
56	Binary (>.5) Metastasis Probability Histogram Kurtosis
57	Binary (<.5) Metastasis Probability Histogram Mean
58	Binary (<.5) Metastasis Probability Histogram Std
59	Binary (<.5) Metastasis Probability Histogram Skew
60	Binary (<.5) Metastasis Probability Histogram Kurtosis
61	High Prob (>=0.9) Stroma Metastasis Probability Histogram Mean
62	High Prob (>=0.9) Stroma Metastasis Probability Histogram Std
63	High Prob (>=0.9) Stroma Metastasis Probability Histogram Skew
64	High Prob (>=0.9) Stroma Metastasis Probability Histogram Kurtosis
65	Count Stroma Metastasis Probability Tiles With Prob >0.5
66	Count Stroma Metastasis Probability Tiles With Prob >=0.9
67	Proportion Stroma Metastasis Probability Tiles With Prob >0.5
68	Proportion Stroma Metastasis Probability Tiles With Prob >=0.9
69	Low Prob (<=0.1) Stroma Metastasis Probability Histogram Mean
70	Low Prob (<=0.1) Stroma Metastasis Probability Histogram Std
71	Low Prob (<=0.1) Stroma Metastasis Probability Histogram Skew
72	Low Prob (<=0.1) Stroma Metastasis Probability Histogram Kurtosis
73	Count Stroma Metastasis Probability Tiles With Prob <0.5
74	Count Stroma Metastasis Probability Tiles With Prob <=0.1
75	Proportion Stroma Metastasis Probability Tiles With Prob <0.5
76	Proportion Stroma Metastasis Probability Tiles With Prob <=0.1
77	Stroma Metastasis Probability Distribution Entrophy
78	# of Met (>=50% probability) Stroma tile clusters
79	# of No Met (>=50% probability) Stroma tile clusters
80	# of 1 to 10 Met (>=50% probability) Stroma tile clusters
81	# of 11 to 20 Met (>=50% probability) Stroma tile clusters
82	# of 21 to 30 Met (>=50% probability) Stroma tile clusters
83	# of >30 Met (>=50% probability) Stroma tile clusters
84	# of No Met (>=50% probability) Stroma tile clusters
85	# of 1 to 5 Met (>=90% probability) Stroma tile clusters
86	# of 6 to 10 Met (>=90% probability) Stroma tile clusters
87	# of >10 Met (>=90% probability) Stroma tile clusters
88	Proportion of No Met (>=50% probability) Stroma tile clusters

89	Proportion of 1 to 10 Met (>=50% probability) Stroma tile clusters
90	Proportion of 11 to 20 Met (>=50% probability) Stroma tile clusters
91	Proportion of 21 to 30 Met (>=50% probability) Stroma tile clusters
92	Proportion of >30 Met (>=50% probability) Stroma tile clusters
93	Proportion of No Met (>=50% probability) Stroma tile clusters
94	Proportion of 1 to 5 Met (>=90% probability) Stroma tile clusters
95	Proportion of 6 to 10 Met (>=90% probability) Stroma tile clusters
96	Proportion of >10 Met (>=90% probability) Stroma tile clusters

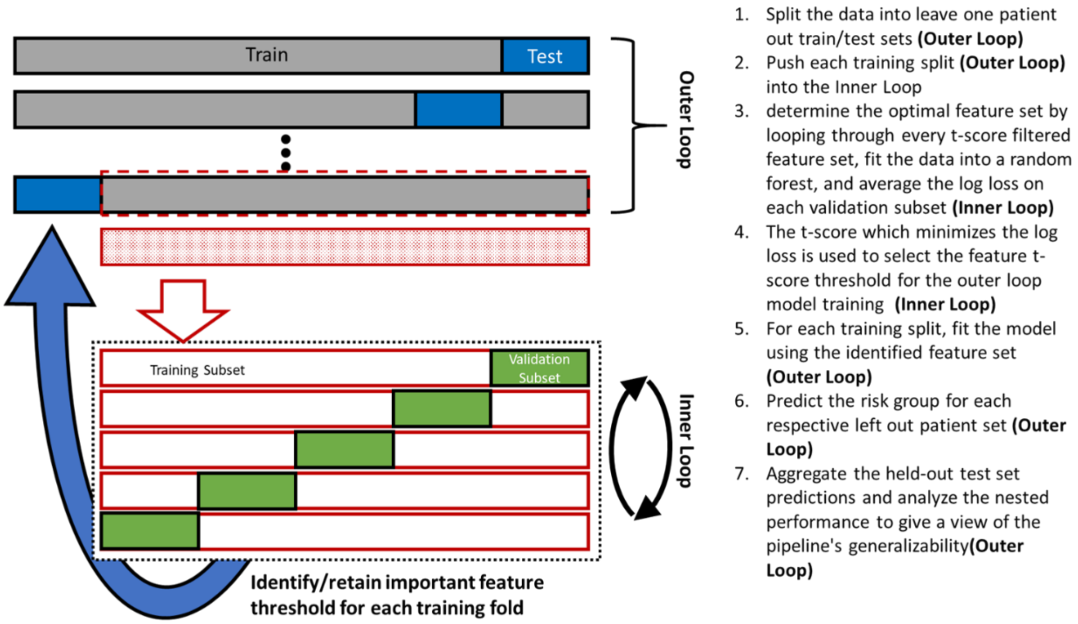

Supplementary Figure 2: Outline of the nested cross validation approach to better test the pipelines generalizability and limit data leakage.

Ground Truth \ Prediction	Cancer	Cancer/Stroma	Fat	Normal	Stroma
Cancer	17,342	256	7	13	1,114
Cancer/Stroma	36	1,268	0	0	31
Fat	0	0	211	0	13
Normal	181	593	16	13,022	484
Stroma	63	291	3	0	8,032

Supplementary Figure 3: Confusion matrix for the validation tiles.

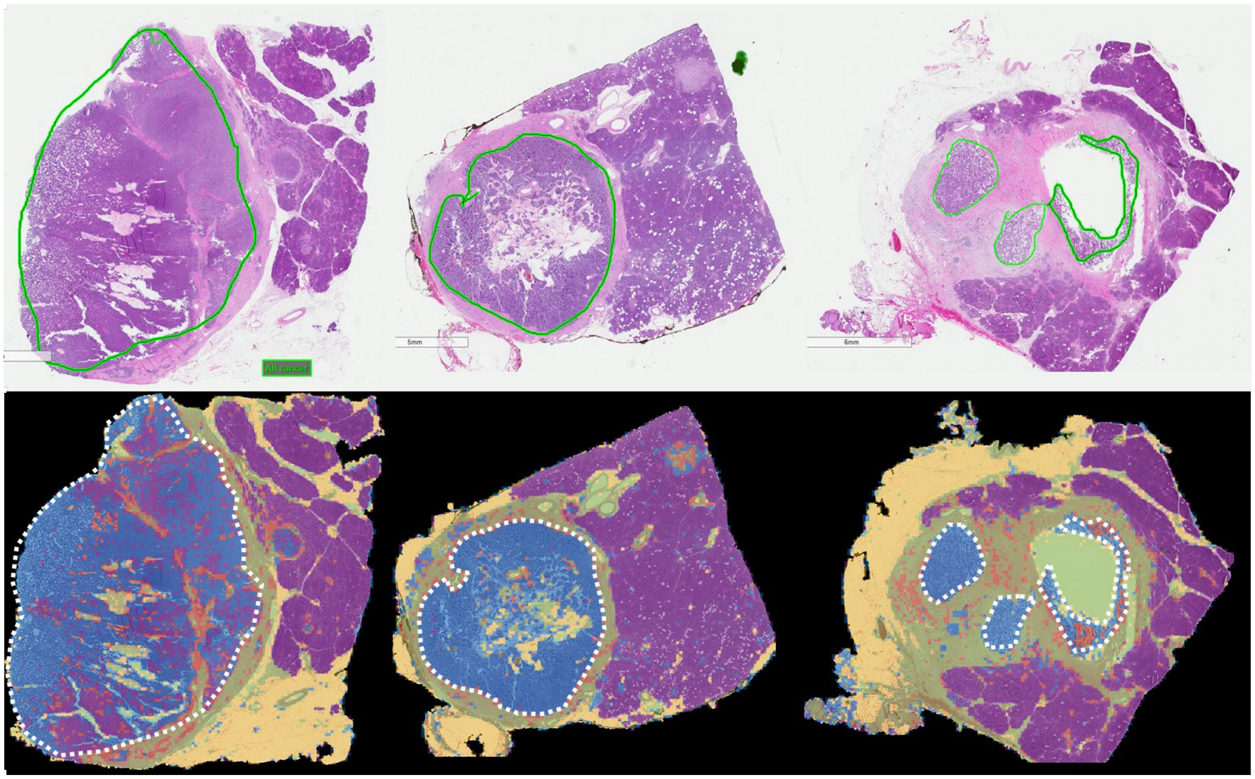

Supplementary Figure 4: Additional examples of representative pathologist-based annotations (solid green line) for cancer regions and automated whole-slide annotation (blue: cancer, red: cancer with stroma, purple: normal parenchymal, green: stroma, yellow: fat, major cancer regions outlined with a white dashed line).

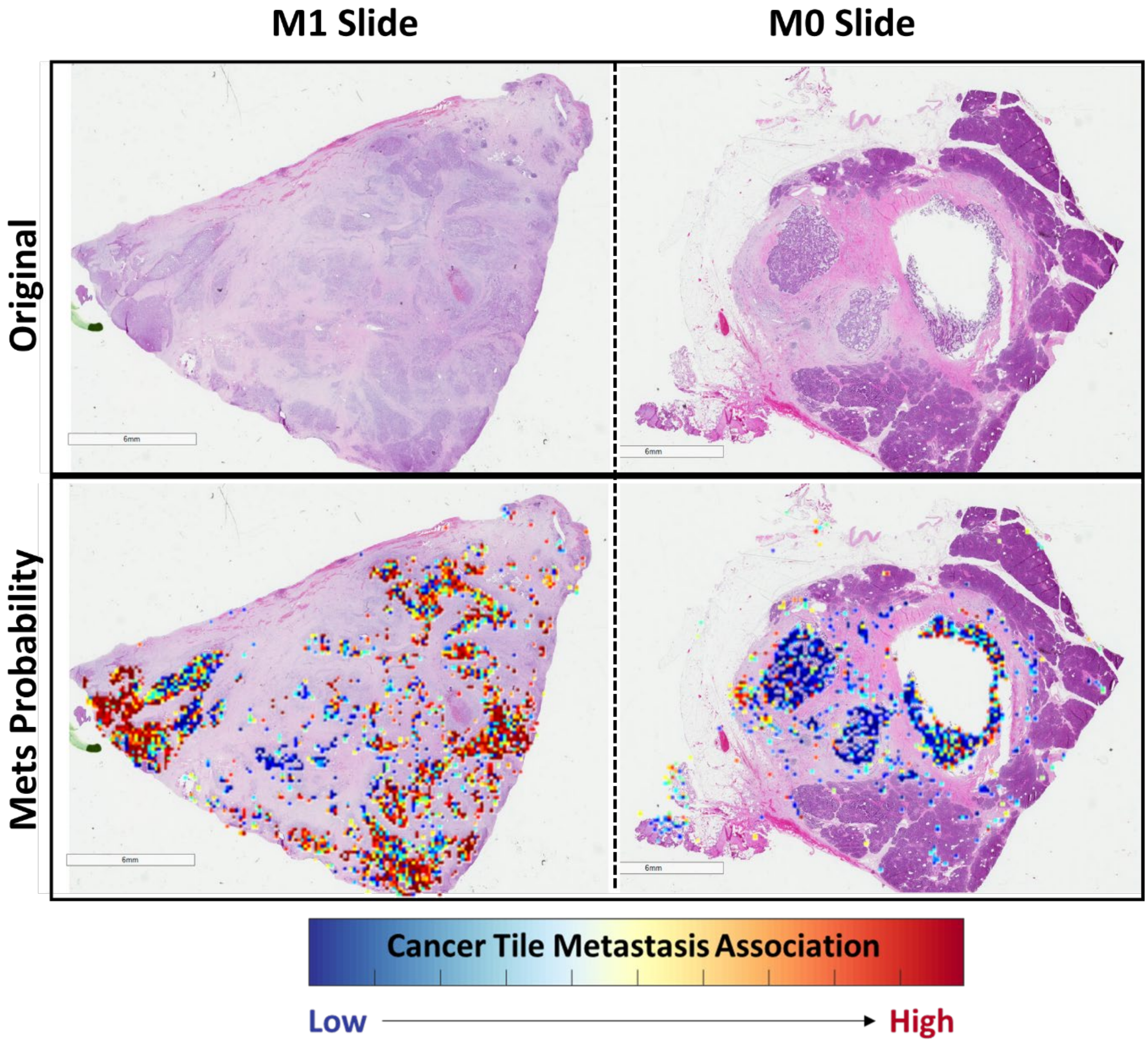

Supplementary Figure 5: Overlayed metastasis probability heatmaps for a slide which came from a patient who metastasized versus one who did not. Blue represents a predicted low metastasis association for the slide and a dark red represent a high metastasis association. M0: no metastasis, M1: metastasis.

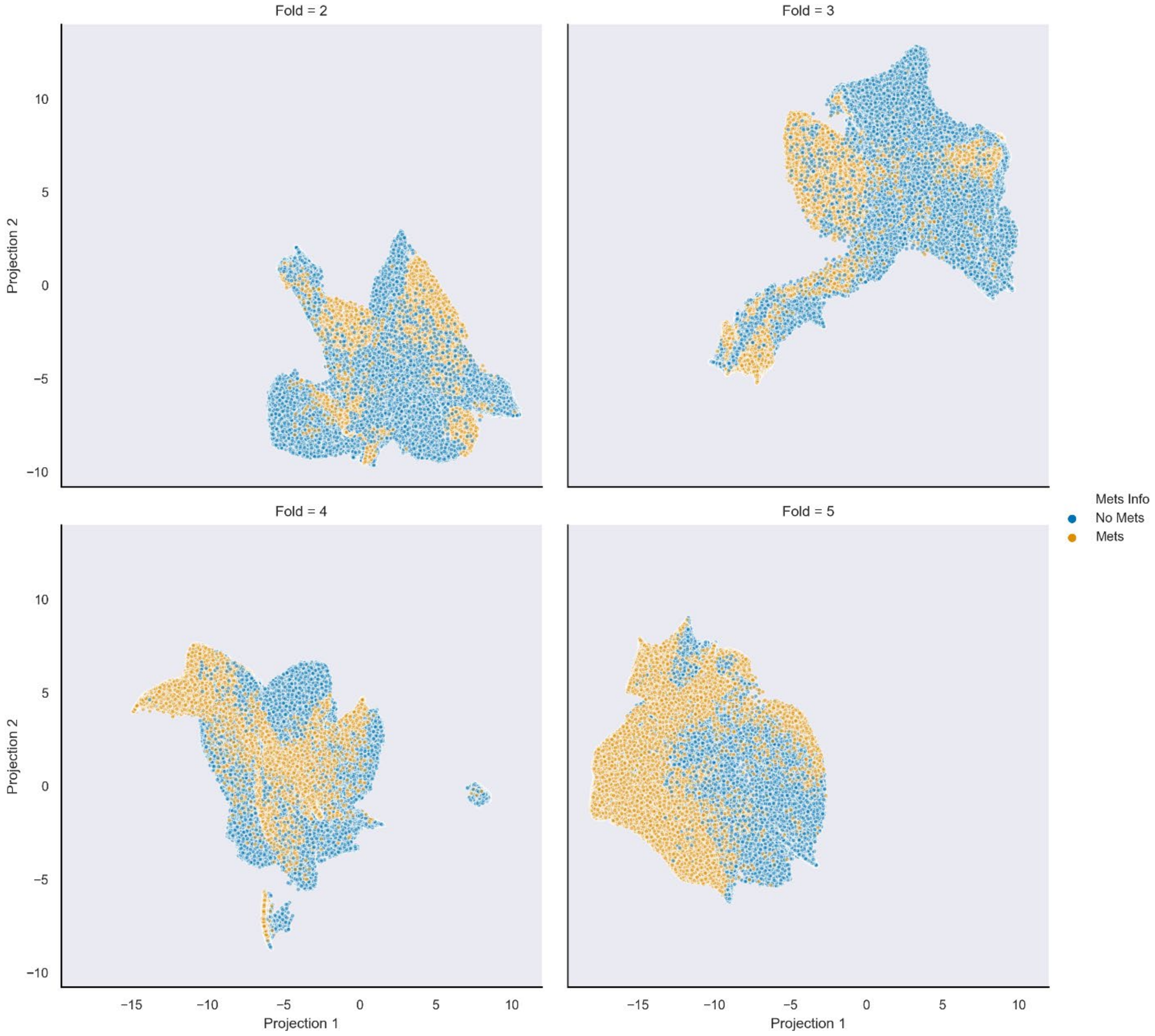

Supplementary Figure 6: UMAP representation, reduced from the activation values of the final CNN pooling layer, for the 2nd-5th fold cancer tiles across 2-dimensions. Each point represents a separate tile with the colors highlighting if the tile came from a patient who metastasized (yellow) versus one who did not (blue).

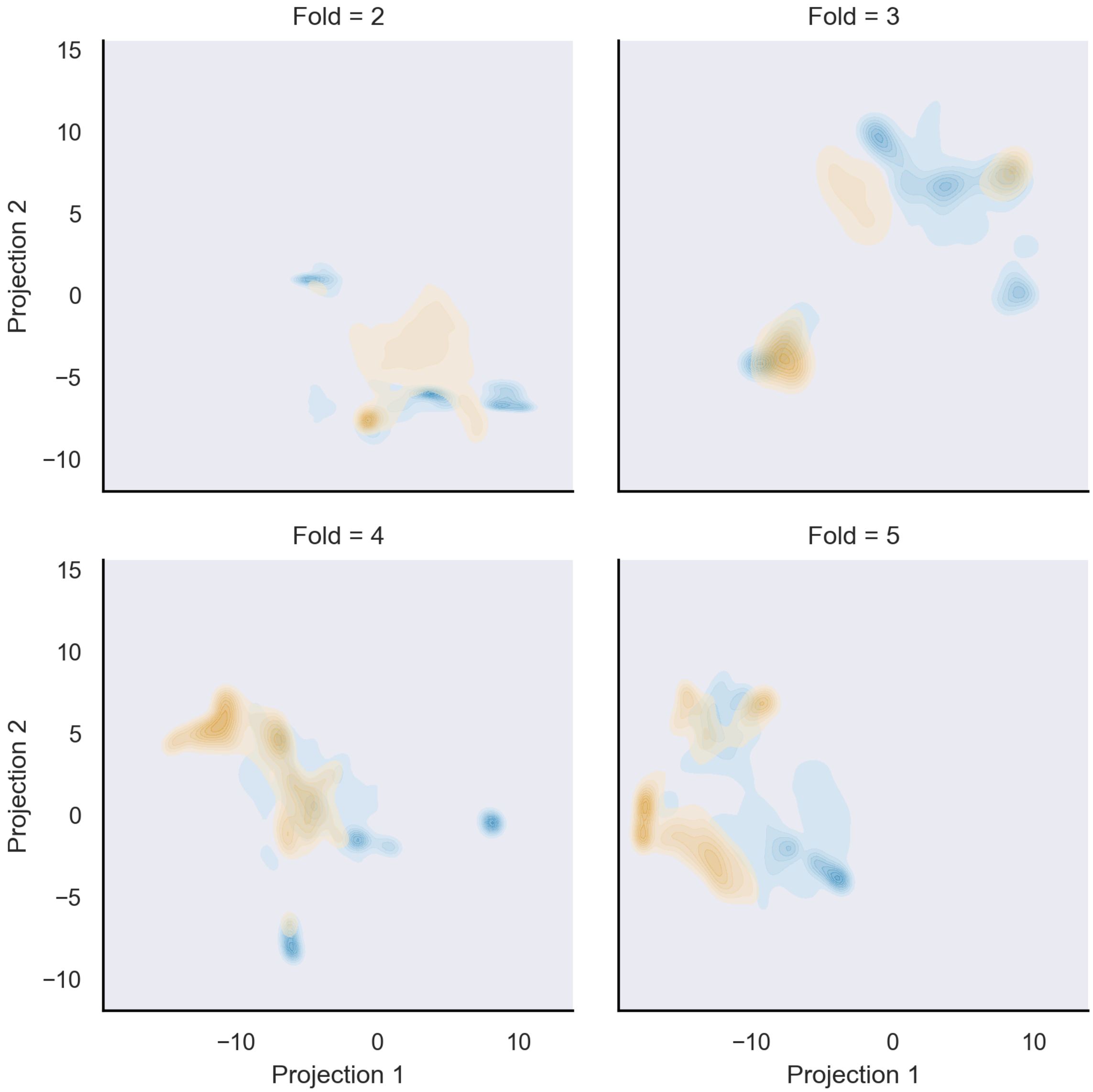

Supplementary Figure 7: UMAP representation density, fit by a KDE, for the 2nd-5th fold cancer tiles across 2-dimensions for each metastatic class.

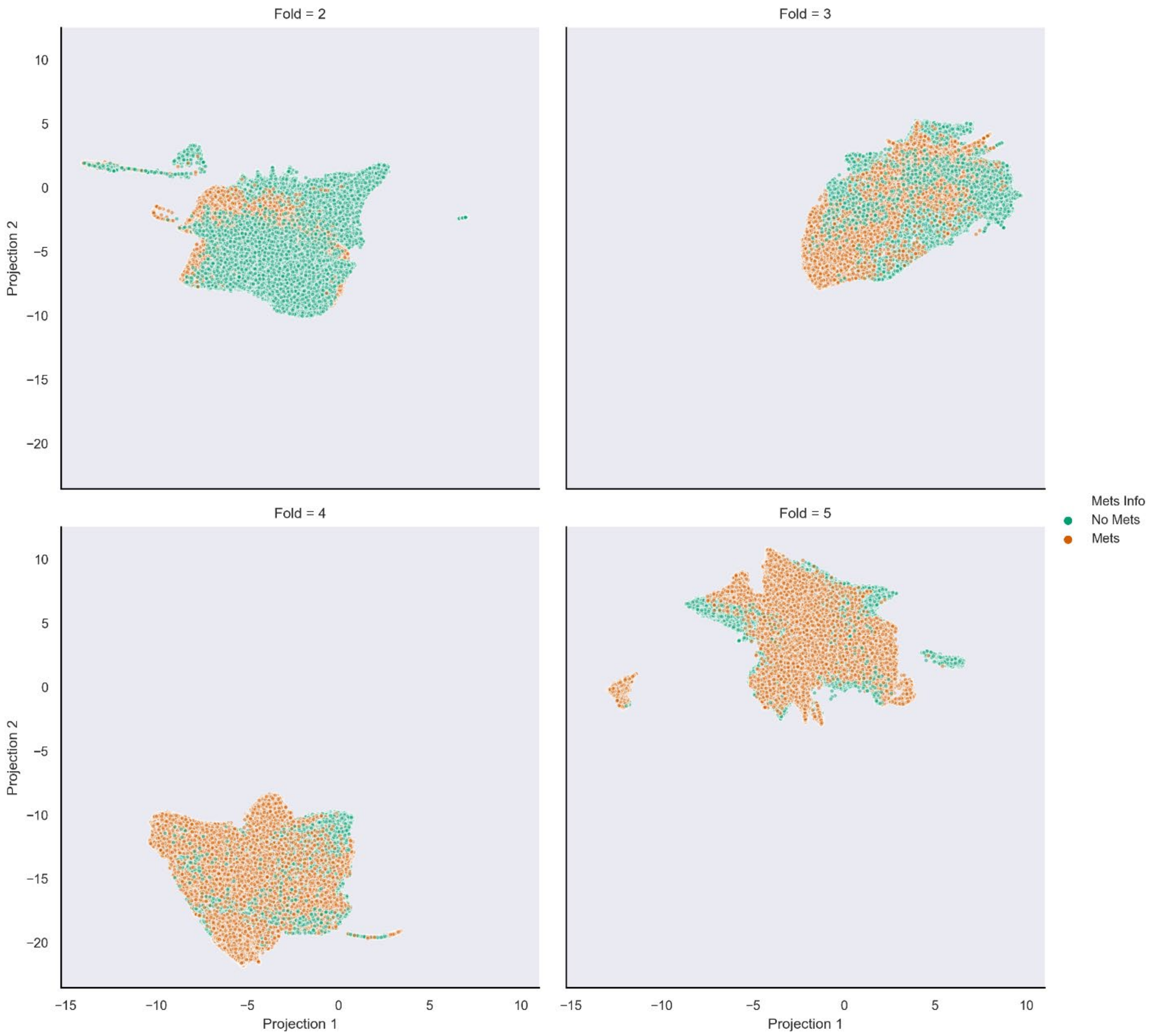

Supplementary Figure 8: UMAP representation, reduced from the activation values of the final CNN pooling layer, for the 2nd-5th fold stroma tiles across 2-dimensions. Each point represents a separate tile with the colors highlighting if the tile came from a patient who metastasized (yellow) versus one who did not (blue).

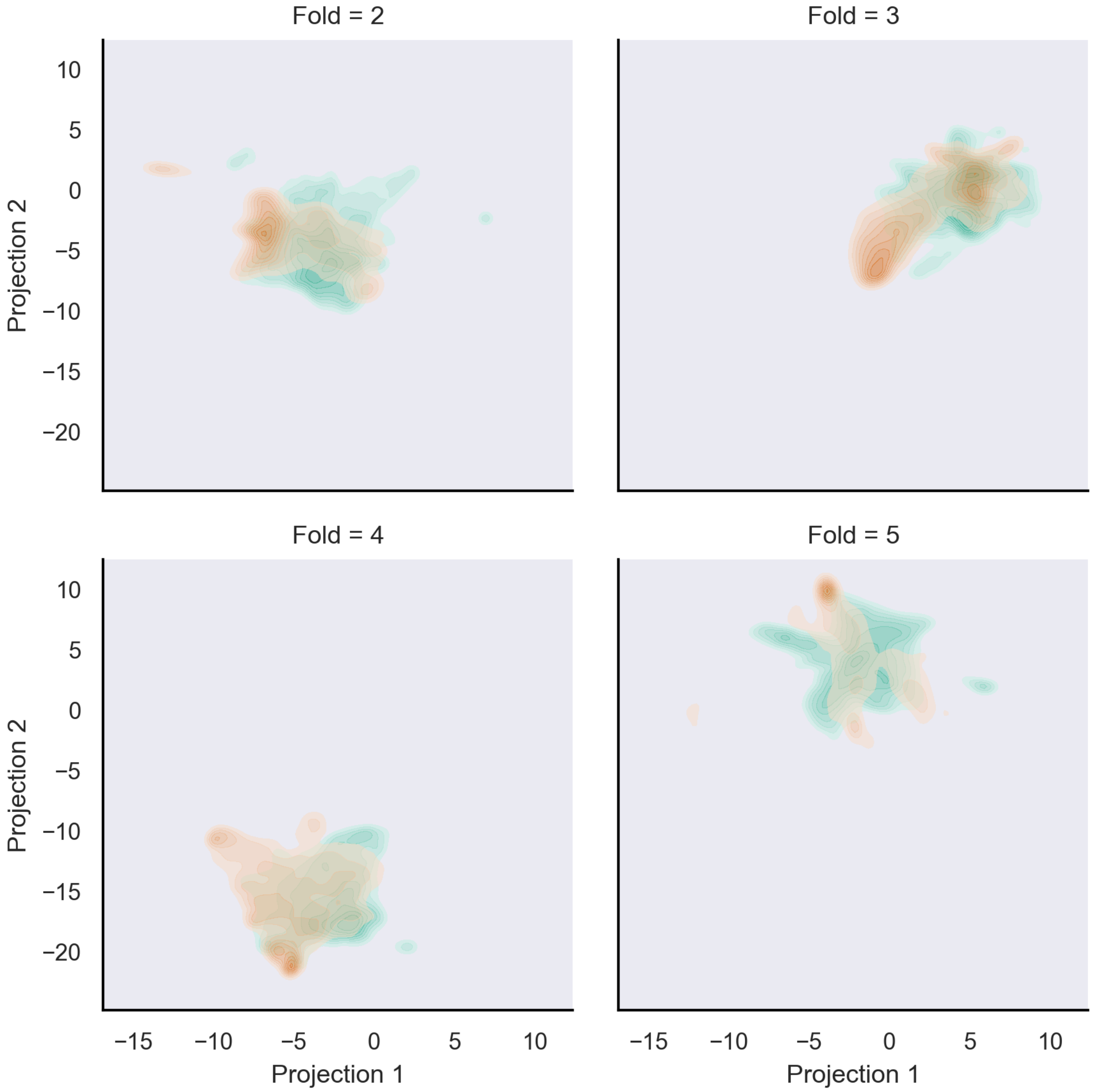

Supplementary Figure 9: UMAP representation density, fit by a KDE, for the 2nd-5th fold stroma tiles across 2-dimensions for each metastatic class.

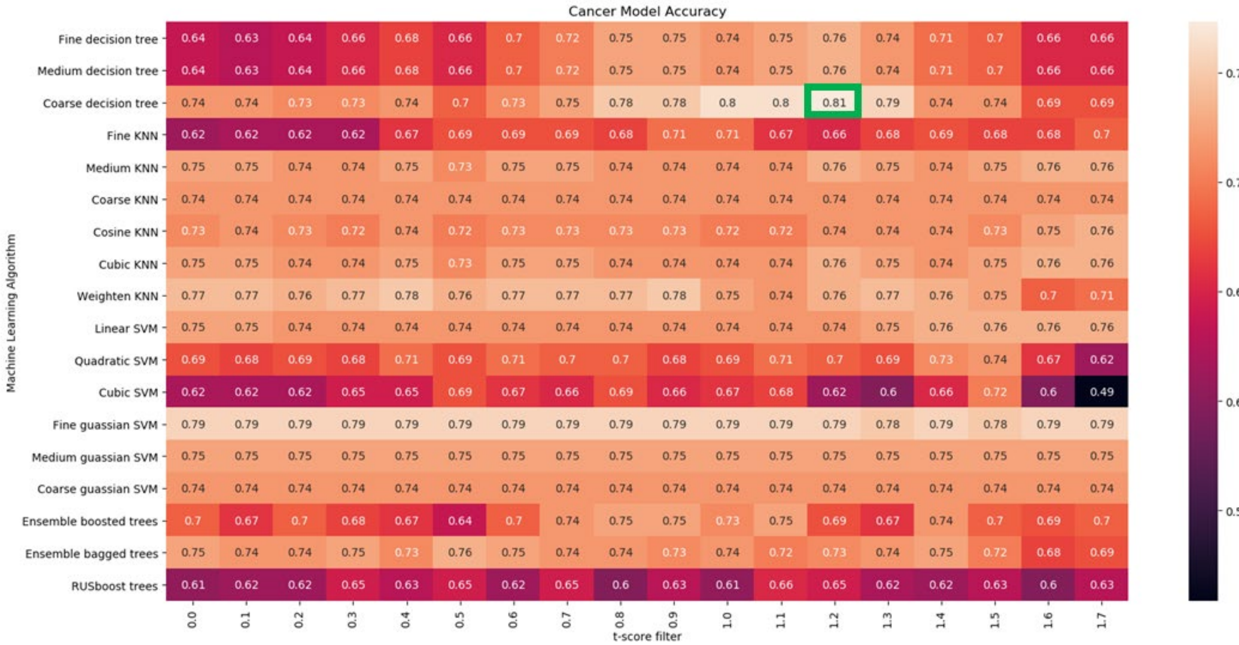

Supplementary Figure 10: Leave-one-out cross validation test set slide accuracy for models built with cancer features. Columns represent t-test filter criteria (i.e. 0.05: remove any features which do not have at least a t-score greater than 0.05 when comparing metastasis status). Best model (green border) is found when using features above a t-score of 1.2 and with a course decision tree.

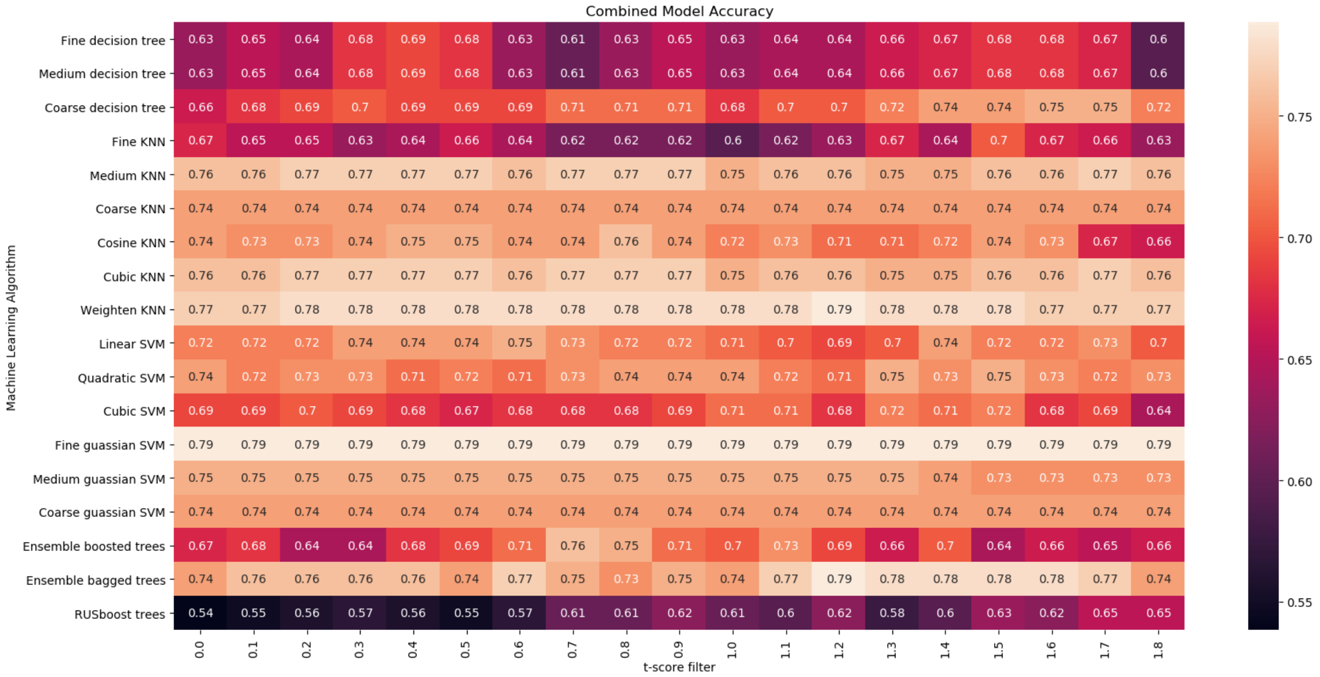

Supplementary Figure 11: Leave-one-out cross validation test set slide accuracy for models built with all whole slide features. Columns represent t-test filter criteria (i.e. 0.05: remove any features which do not have at least a t-score greater than 0.05 when comparing metastasis status).

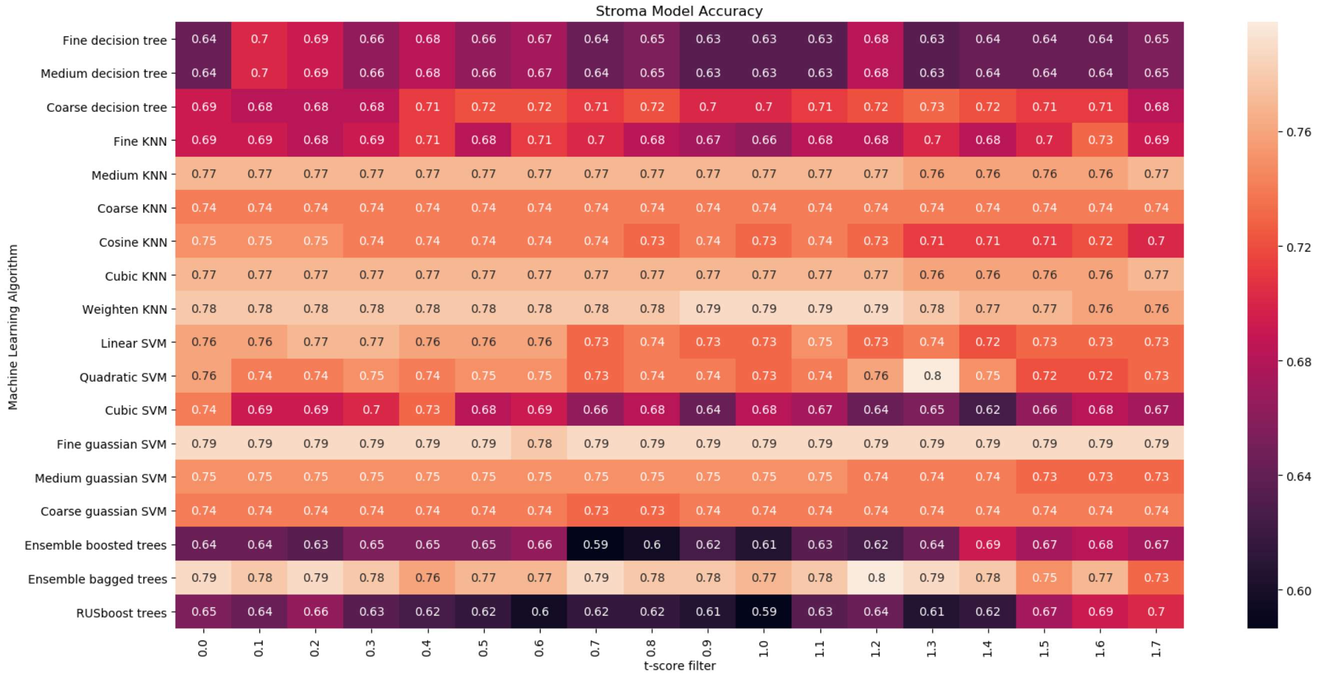

Supplementary Figure 12: Leave-one-out cross validation test set slide accuracy for models built with stroma slide features. Columns represent t-test filter criteria (i.e. 0.05: remove any features which do not have at least a t-score greater than 0.05 when comparing metastasis status).

Supplementary Table 4: Confusion matrix and performance metrics for both the slide and patient levels for the final model for the full and low-grade cohorts.

Confusion Matrix

Level	Cohort	Cancer Model	Ground Truth: No Mets.	Ground Truth: Mets.
Slide Level	*All Slides*	Low Risk	73	16
		High Risk	4	11
	Low Grade (I/II)	Low Risk	61	13
		High Risk	2	6
Patient Level	*All Slides*	Low Risk	67	9
		High Risk	4	9
	Low Grade (I/II)	Low Risk	55	7
		High Risk	2	5

Performance Metrics

	Slide Level: *All Slides*	Slide Level: *Low Grade (I/II)*	Patient Level: *All Slides*	Patient Level: *Low Grade (I/II)*
Accuracy:	0.8077	0.8171	0.8539	0.8696
Sensitivity:	0.4074	0.3158	0.5000	0.4167
Specificity:	0.9481	0.9683	0.9437	0.9649
Precision:	0.7333	0.7500	0.6923	0.7143
Negative Predictive Value:	0.8202	0.8243	0.8816	0.8871
F1 score:	0.5238	0.4444	0.5806	0.5263
Balanced Accuracy:	0.6777	0.6420	0.7218	0.6908
Matthews Corr. Coef.:	0.4436	0.4039	0.5046	0.4790

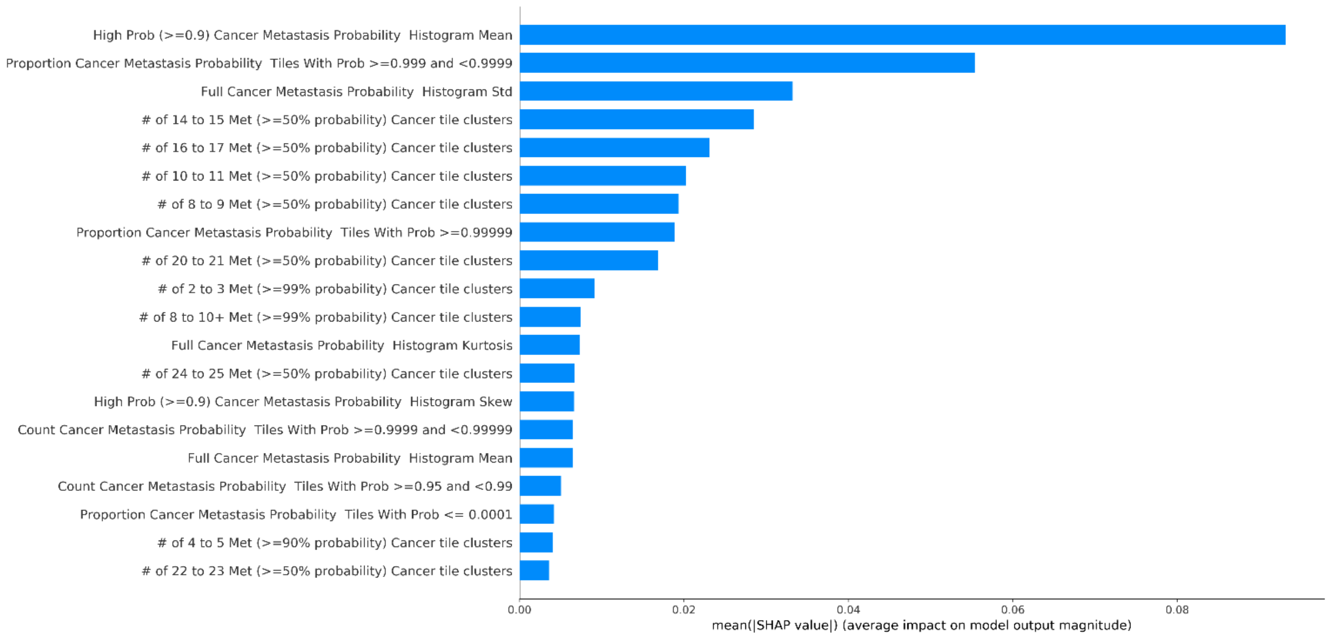

Supplementary Figure 13: Feature importance of the final, cancer tile based, model based on LOOCV SHAP analysis.

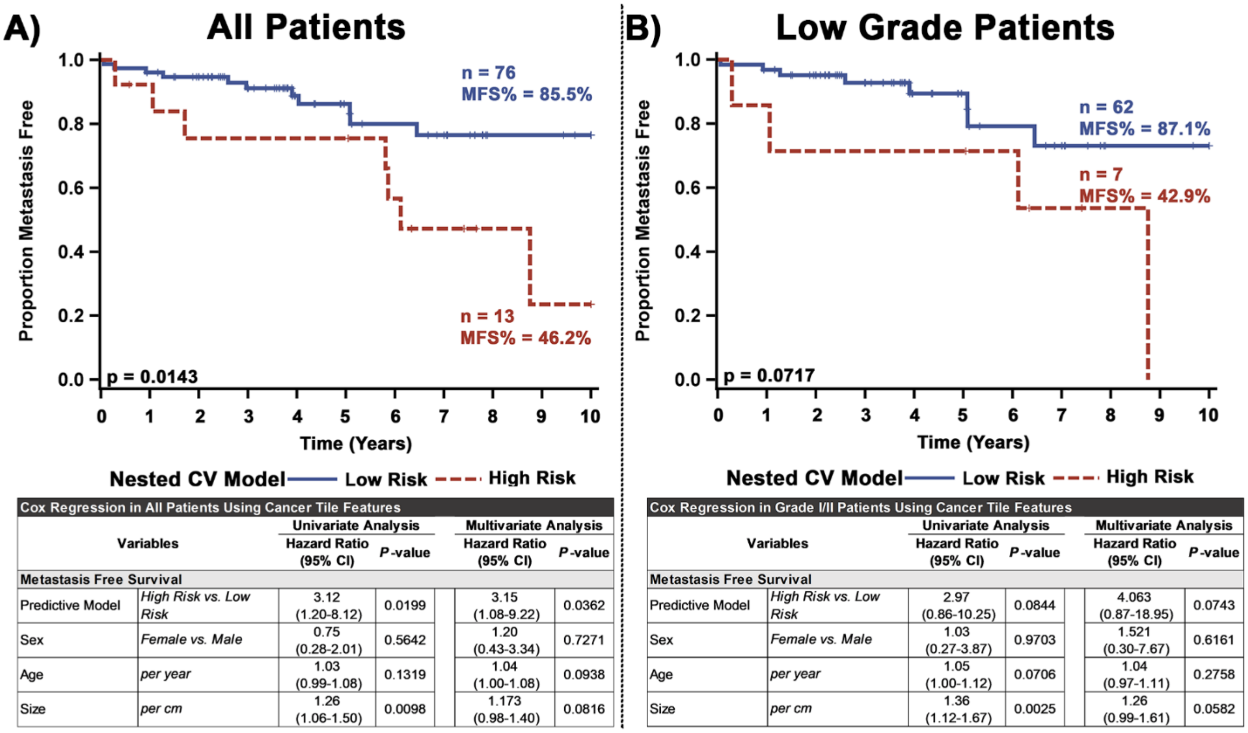

Cox Regression in All Patients Using Cancer Tile Features					
Variables		Univariate Analysis		Multivariate Analysis	
		Hazard Ratio (95% CI)	*P*-value	Hazard Ratio (95% CI)	*P*-value
Metastasis Free Survival					
Predictive Model	*High Risk vs. Low Risk*	3.12 (1.20-8.12)	0.0199	3.15 (1.08-9.22)	0.0362
Sex	*Female vs. Male*	0.75 (0.28-2.01)	0.5642	1.20 (0.43-3.34)	0.7271
Age	*per year*	1.03 (0.99-1.08)	0.1319	1.04 (1.00-1.08)	0.0938
Size	*per cm*	1.26 (1.06-1.50)	0.0098	1.173 (0.98-1.40)	0.0816

Cox Regression in Grade I/II Patients Using Cancer Tile Features					
Variables		Univariate Analysis		Multivariate Analysis	
		Hazard Ratio (95% CI)	*P*-value	Hazard Ratio (95% CI)	*P*-value
Metastasis Free Survival					
Predictive Model	*High Risk vs. Low Risk*	2.97 (0.86-10.25)	0.0844	4.063 (0.87-18.95)	0.0743
Sex	*Female vs. Male*	1.03 (0.27-3.87)	0.9703	1.521 (0.30-7.67)	0.6161
Age	*per year*	1.05 (1.00-1.12)	0.0706	1.04 (0.97-1.11)	0.2758
Size	*per cm*	1.36 (1.12-1.67)	0.0025	1.26 (0.99-1.61)	0.0582

Supplementary Figure 14: Univariate and multivariate analyses of the nested cross validated PanNET metastasis risk prediction pipeline (aggregated LOOCV test sets) for (A) all patients and (B) Low grade (I/II) patients.
