# Supplementary figures and images for: Predicting Metastasis Risk in Pancreatic Neuroendocrine Tumors Using Deep Learning Image Analysis
Source: Front Oncol. 2021 Feb 25;10:593211. doi: 10.3389/fonc.2020.593211 (PMC7946991; doi:10.3389/fonc.2020.593211)

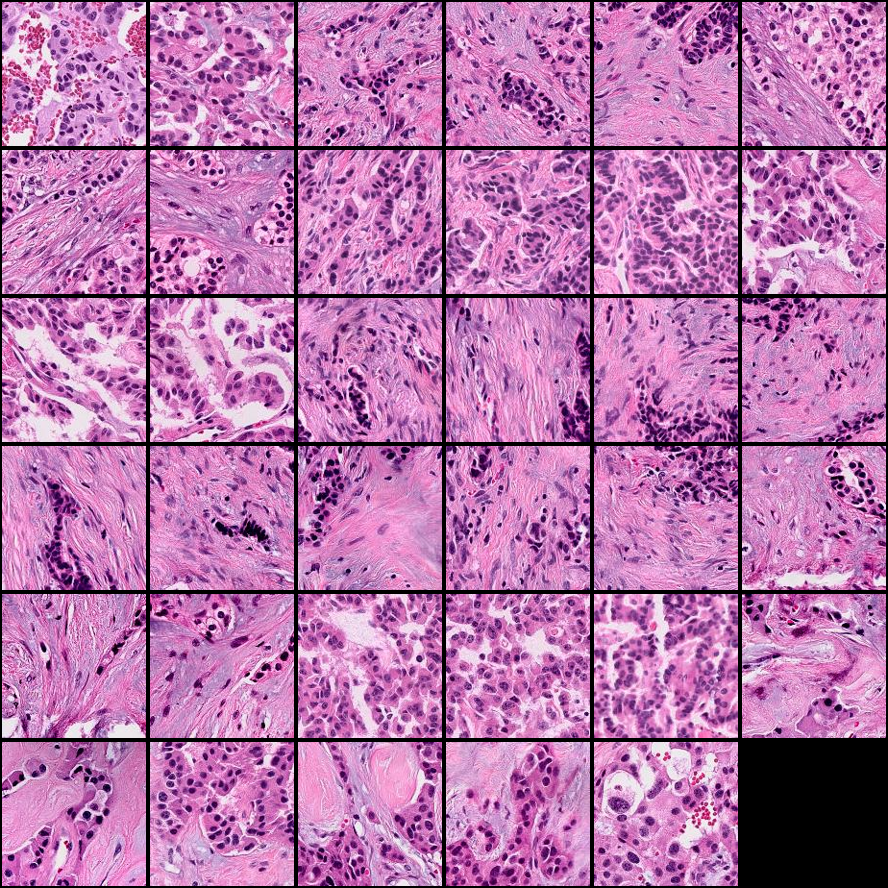

Supplement: Supplementary Data sheet 1 — Montages of tiles found to be a high/low metastasis risk and with/without future metastasis. [file DataSheet_1.zip › HighRisk_And_Mets.tif]

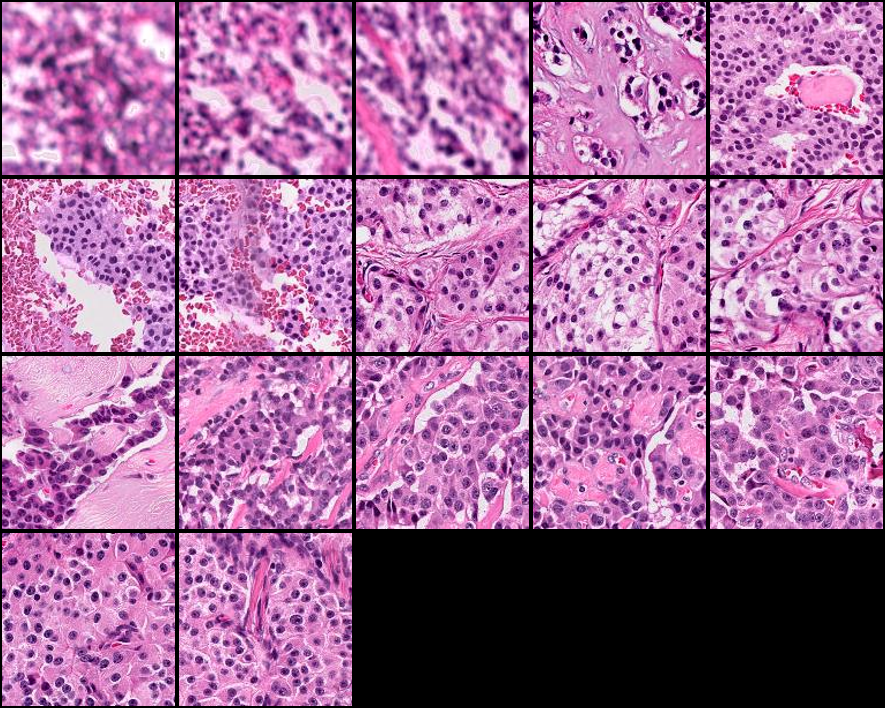

Supplement: Supplementary Data sheet 1 — Montages of tiles found to be a high/low metastasis risk and with/without future metastasis. [file DataSheet_1.zip › HighRisk_And_NoMets.tif]

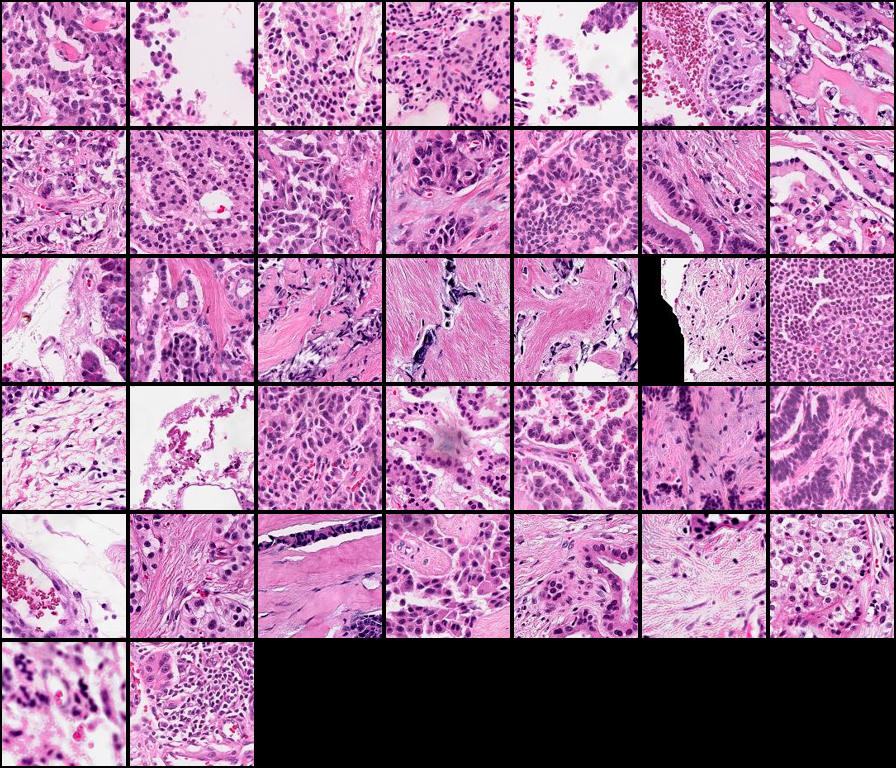

Supplement: Supplementary Data sheet 1 — Montages of tiles found to be a high/low metastasis risk and with/without future metastasis. [file DataSheet_1.zip › LowRisk_And_Mets.tif]

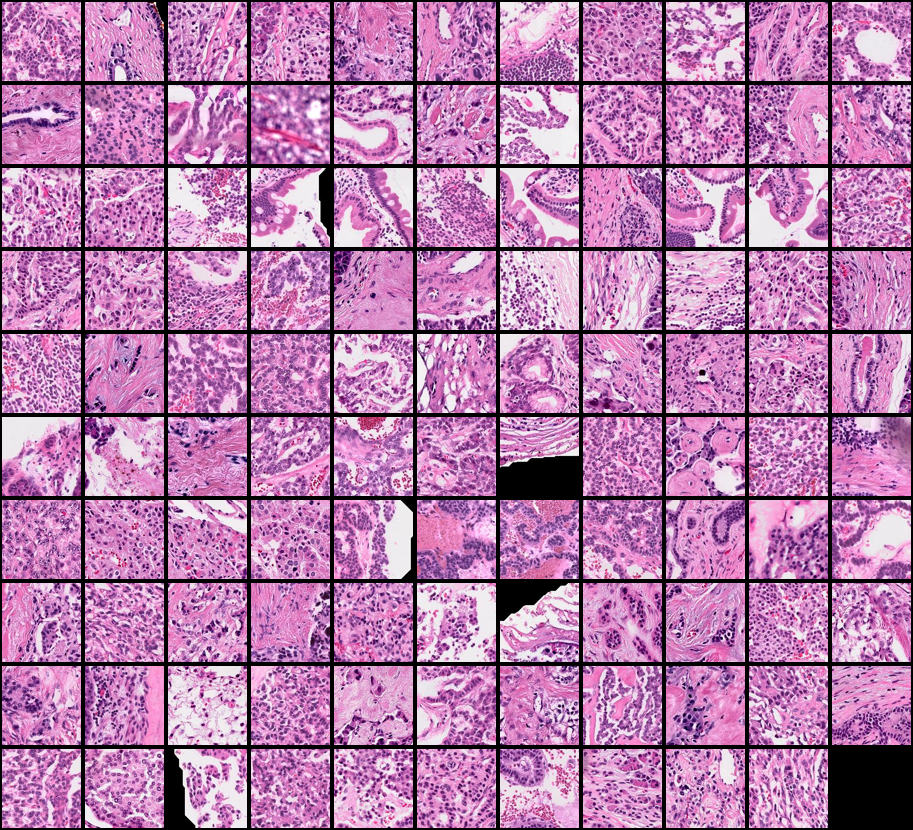

Supplement: Supplementary Data sheet 1 — Montages of tiles found to be a high/low metastasis risk and with/without future metastasis. [file DataSheet_1.zip › LowRisk_And_NoMets.tif]
